# Amplitudes and time scales of picosecond-to-microsecond motion in proteins studied by solid-state NMR: a critical evaluation of experimental approaches and application to crystalline ubiquitin

**DOI:** 10.1007/s10858-013-9787-x

**Published:** 2013-10-09

**Authors:** Jens D. Haller, Paul Schanda

**Affiliations:** 1 Univ. Grenoble Alpes, Institut de Biologie Structurale (IBS), 38027 Grenoble, France; 2 CEA, DSV, IBS, 38027 Grenoble, France; 3 CNRS, IBS, 38027 Grenoble, France

**Keywords:** Solid-state NMR, Protein dynamics, Dipolar couplings, REDOR, Model-free, Ubiquitin, Order parameters, Relaxation

## Abstract

Solid-state NMR provides insight into protein motion over time scales ranging from picoseconds to seconds. While in solution state the methodology to measure protein dynamics is well established, there is currently no such consensus protocol for measuring dynamics in solids. In this article, we perform a detailed investigation of measurement protocols for fast motions, i.e. motions ranging from picoseconds to a few microseconds, which is the range covered by dipolar coupling and relaxation experiments. We perform a detailed theoretical investigation how dipolar couplings and relaxation data can provide information about amplitudes and time scales of local motion. We show that the measurement of dipolar couplings is crucial for obtaining accurate motional parameters, while systematic errors are found when only relaxation data are used. Based on this realization, we investigate how the REDOR experiment can provide such data in a very accurate manner. We identify that with accurate rf calibration, and explicit consideration of rf field inhomogeneities, one can obtain highly accurate absolute order parameters. We then perform joint model-free analyses of 6 relaxation data sets and dipolar couplings, based on previously existing, as well as new data sets on microcrystalline ubiquitin. We show that nanosecond motion can be detected primarily in loop regions, and compare solid-state data to solution-state relaxation and RDC analyses. The protocols investigated here will serve as a useful basis towards the establishment of a routine protocol for the characterization of ps–μs motions in proteins by solid-state NMR.

**Electronic supplementary material:**

The online version of this article (doi:10.1007/s10858-013-9787-x) contains supplementary material, which is available to authorized users.

## Introduction

The three-dimensional structure that a protein spontaneously adopts in its environment is dictated by a subtle balance of numerous interactions, which are all individually weak. At physiologically relevant temperatures, these interactions are continuously rearranged, allowing a protein to dynamically sample a range of different conformational states. The dynamic processes that connect these various conformational states on the complex energy landscape of a protein take place on a wide range of time scales. Elucidating the interconversions between these various states is crucial for the understanding of biomolecular function at atomic level. Characterizing protein motion at an atomic scale is a challenging task, as it requires, in principle, the determination of a multitude of structures, their relative energies as well as the time scales (and thus, energy barriers) that link these states. Relevant time scales for dynamic biomolecular processes cover over twelve orders of magnitude (ps–s), a breadth that represents a severe challenge to any experimental method.

Solution-state NMR is a very well established method to address protein dynamics at atomic resolution. A number of solution-state NMR approaches exist to study motion on time scales from picoseconds to minutes (Kleckner and Foster 2011; Mittermaier and Kay 2009; Palmer 2004). The mobility of proteins on short time scales, from picoseconds to microseconds, corresponds to interconversion between structurally similar states separated by low energy barriers. This fast protein motion is the focus of the present paper. Most often, the breadth of the conformational space sampled on this time scale is expressed in the simplified terms of an order parameter, S^2^ (Lipari and Szabo 1982a), or, equivalently, fluctuation opening angle (Brüschweiler and Wright 1994) that describes the motional freedom of a given bond vector under consideration; the corresponding time scale of the fluctuations is expressed as correlation time, τ. Alternative to these approaches, the “slowly relaxation local structure” approach has also been employed to study ps-ns motion in proteins (Meirovitch et al. 2010).

Although these sub-microsecond time scale motions are generally much faster than actual functional turnover rates in proteins (e.g. enzymatic reactions or folding rates), the fast local motions may be functionally relevant as they are thought to contribute to stability and facilitate ligand-binding through the entropic contributions (Frederick et al. 2007; Yang and Kay 1996). Therefore, the determination of sub-microsecond motions is of considerable interest, and is routinely performed in solution-state NMR. In order to be able to decipher the above-mentioned entropy–motion relationship, it is crucial that the motional amplitudes can be determined with high accuracy, i.e. that systematic biases are eliminated. For the case of solution-state NMR, the measurement of ^15^N relaxation is the established way to measure backbone mobility on time scales up to a few nanoseconds, the time scale of overall molecular tumbling. Provided some experimental care (Ferrage et al. 2009), these experimental approaches provide quantitative measures of motion, and, thus, can be translated e.g. to entropy (Yang and Kay 1996). The interpretation of dynamics data from NMR can also be guided and assisted through MD simulations, which allow getting a mechanistic insight. (Granata et al. 2013; Xue et al. 2011).

In recent years, solid-state NMR (ssNMR) spectroscopy has evolved into a mature method for studying protein structure, interactions, and dynamics in biological systems that are unsuited for solution-state NMR, such as insoluble aggregates or very large assemblies. In the context of fast (ps to μs) motions, solid-state NMR may be significantly more informative than its solution-state counterpart, as the time scale above a few nanoseconds—invisible in solution-state NMR because of the overall molecular tumbling—is readily accessible. In contrast to solution state NMR, to date there is no consensus protocol about the methodology for measuring motions by ssNMR. In the solid state, several routes are possible to address fast motions (ps–μs). (1) Spin relaxation is sensitive to both time scales and amplitudes and, in the case of ^15^N spins, can be measured and interpreted in a rather straightforward manner, as the relaxation is largely dominated by the dipole interaction to the attached ^1^H and the ^15^N CSA. Approaches for measuring longitudinal (Chevelkov et al. 2008; Giraud et al. 2004) (R_1_) and transverse (Chevelkov et al. 2007; Lewandowski et al. 2011) (R_1ρ_ and cross-correlated) relaxation parameters in proteins have been proposed. (2) Measuring the motion-induced reduction of anisotropic interactions (dipolar couplings, chemical shift anisotropies) provides direct access to the amplitude of all motions occurring on time scales up to the inverse of the interaction strength (in the kHz range), through the reduction of the coupling values from the rigid-limit values; the case of the dipolar coupling of directly bonded nuclei is most attractive, as the rigid-limit value of the interaction is readily computed from the bond length. In principle, the measurement of site-specific CSA tensors may confer similar information (Yang et al. 2009), although the interpretation is more difficult because the static-limit CSA is not easily determined.

Different approaches have been proposed in recent studies of protein dynamics, as to which type of the above data should be used for determination of motional parameters, as well as to how these experimental data should be acquired (Chevelkov et al. 2009a, b; Lewandowski et al. 2011; Schanda et al. 2010; Yang et al. 2009), and even whether they should be interpreted in terms of local or global motion (Lewandowski et al. 2010a). In this manuscript, we systematically investigate ways to determine backbone dynamics in proteins using various longitudinal and transverse ^15^N relaxation rates, as well as ^1^H–^15^N dipolar coupling measurements. We show that ^15^N relaxation data are generally insufficient to correctly describe amide backbone dynamics, even when different types of relaxation rate constants are measured at multiple static magnetic field strengths. In particular, relaxation data fail to correctly report on picosecond motion. We find that only the addition of ^1^H–^15^N dipolar couplings allows resolving this problem. We investigate in detail how systematic errors in such dipolar-coupling measurements can arise, using the REDOR scheme, and show how they are suppressed to below 1 %. Together with the relaxation analysis, this study will serve as a useful guide for analysis of protein backbone motion by solid-state NMR.

We report new NH dipolar coupling measurements and ^15^N R_1ρ_ data, measured on a microcrystalline preparation of deuterated ubiquitin at MAS frequencies of 37–40 kHz. Together with previously reported relaxation data (a total of up to 7 data points per residue), we investigate backbone mobility in microcrystalline ubiquitin, and compare the results to solution-state NMR data.

## Materials and methods

In addition to previously reported relaxation data on microcrystalline ubiquitin (Schanda et al. 2010), we have measured ^15^N R_1ρ_ relaxation rate constants and ^1^H–^15^N dipolar couplings. All experimental data reported were collected on a Agilent 600 MHz VNMRS spectrometer equipped with a triple-resonance 1.6 mm Fast-MAS probe tuned to ^1^H, ^13^C and ^15^N. A microcrystalline sample of u-[^2^H^13^C^15^N]-labeled ubiquitin, back-exchanged to ^1^H at 50 % of the exchangeable sites was prepared as described previously (Schanda et al. 2010, 2009). ^15^N R_1ρ_ relaxation rates were measured at a MAS frequency of 39.5 kHz. The ^15^N spin lock field strength was set to 15 kHz and the R_1ρ_ decay was monitored by incrementing the spin lock duration from 5 to 250 ms (total 10 points).

^1^H–^15^N dipolar couplings were measured at 37.037 kHz MAS frequency. In all cases, the effective sample temperature was kept at 300 K, as determined from the bulk water resonance frequency. MAS frequencies were stable to within <10 Hz.

All NMR spectra were proton-detected; the pulse sequence for the ^1^H–^15^N dipolar coupling measurement is shown in Fig. 3, and the experiment for R_1ρ_ is similar, with the REDOR element being replaced by a ^15^N spin lock of variable duration.

All NMR data were processed with nmrPipe (Delaglio et al. 1995), and analyzed with NMRview (OneMoon Scientific. Inc.). Peak volumes were obtained by summing over rectangular boxes; error estimates on the volumes were calculated from the square root of the number of summed points multiplied by three times the standard deviation of the spectral noise.

For the analysis of the ^1^H–^15^N dipolar coupling measurement experiment, in-house written GAMMA (Smith et al. 1994) simulation programs were used, and dipolar couplings were obtained using a grid-search strategy, as previously described (Schanda et al. 2010).

All data analyses, i.e. the fitting of the dipolar couplings, as well as the fit of R_1ρ_ relaxation curves and the model-free analyses were performed with in-house written python programs. Relaxation rate constants for R_1_ and the dipolar-CSA cross-correlated relaxation rate constants (η) were calculated as described before (Schanda et al. 2010); the R_1ρ_ rates were converted to R_2_ via the chemical shift offset and the R_1_, as

1 $$ {\text{R}}_{2} = \left[ {{\text{R}}_{1\rho } -{\text{R}}_{1} \cos^{2} \left( \theta \right)} \right]/\sin^{2} \left( \theta \right) $$

where θ is the angle between the effective spin-lock field and the external magnetic field (90° represents a resonance exactly on-resonance with the spin-lock field). These corrected R_2_ rate constants are essentially identical to the measured R_1ρ_, because θ is close to 90° for almost all residues (average: 88.6°, minimal value 85°), and R_1_ is very small compared to R_1ρ_.

The R_2_ rate constant is given as:

2 $$ {\text{R}}_{2} =\, \left( {1/20} \right){\text{d}}^{2} \left( {4{\text{J}}\left( 0 \right) + {\text{J}}\left( {\omega_{\text{H}} - \omega_{\text{N}} } \right) + 3{\text{J}}\left( {\omega_{\text{N}} } \right) +\, 6{\text{J}}\left( {\omega_{\text{H}} } \right) + 6{\text{J}}\left( {\omega_{\text{H}} + \omega_{\text{N}} } \right)} \right) + 1/15{\text{c}}^{2} {\text{J}}\left( {\omega_{\text{N}} } \right) + 4/45{\text{ c}}^{2} {\text{J}}\left( 0 \right) $$

where all constants are defined as in (Schanda et al. 2010). Equivalent expressions for R_1_ and the cross-correlated relaxation rate constant are also given there.

Spectral densities, J(ω) were computed according to the simple model-free (SMF) or extended model-free (EMF) approach, as

3 $$ J(\omega ) = (1 - S^{2} )\frac{\tau }{{1 + \omega^{2} \tau^{2} }} $$

for SMF and

4 $$ J(\omega ) = \left(1 - S_{f}^{2}\right)\frac{\tau }{{1 + \omega^{2} \tau_{f}^{2} }} + S_{f}^{2} \left(1 - S_{s}^{2} \right)\frac{\tau }{{1 + \omega^{2} \tau_{s}^{2} }} $$

for EMF. In EMF, fast and slow motional contributions are denoted with the subscripts “f” and “s”, respectively.

In all analyses a N-H bond length of 1.02 Å was used (Bernado and Blackledge 2004), and the ^15^N CSA was assumed to be axially symmetric with σ_z_ = 113 ppm (Δσ = 170 ppm). The N-H bond length may vary slightly across the sequence, as a consequence of hydrogen bonding of amides. Particularly, it might be that amides in secondary structure elements have longer N–H bonds. This would lead to a decrease in the measured dipolar couplings. We assume that this effect is minor, because we find that the measured dipolar couplings in secondary structure elements are higher than in loops, which is the opposite of what is expected if bond elongation was dominant. Furthermore, we (Schanda et al. 2010) and others (Chevelkov et al. 2009b) also showed previously that there is no correlation between the dipolar coupling and the amide chemical shift (which, in turn, correlates with the H-bond strength). If our assumption of uniform bond length was incorrect, i.e. if NH bonds were longer in secondary structures than in loops, then the order parameters that we report would slightly underestimate the real values in secondary structures, and overestimate the values in loop regions. As explained above, we believe that it is safe to neglect these effects.

^15^N CSA tensors may vary from site to site, as a consequence of structural differences between different peptide planes. As a consequence, the relaxation rates are impacted, because the CSA is one of the two relaxation mechanisms that relax the ^15^N spin. Note that the CSA mechanism is generally the less important one compared to the dipolar interaction, in particular given the fact that our study was performed at a rather low field strength where the CSA is small (in Hertz).

Note that the site-to-site variation of the ^15^N CSA tensor has only a very small effect on the determination of ^1^H–^15^N dipolar couplings using the REDOR experiment (Schanda et al. 2011b). Likewise, the ^1^H CSA tensor has negligible effects on the apparent dipolar coupling, see (Schanda et al. 2011b) and Figure S9 in the Supporting Information.

Best-fit parameters in the two different model-free models were obtained by minimizing the target functions:

5 $$ \chi^{2} = \sum\limits_{i} {\frac{{(X_{i,calc} - X_{i,\exp }  )^{2}}}{{\sigma_{i,\exp }^{2} }}} $$

where *X*_i_ are the observables (R_1_, R_2_, η or dipolar order parameter S^2^), σ_exp_ is the experimental error margin on the parameter *X*.

As described in the text, we also used fits where the order parameter was fixed to the dipolar-coupling derived one. Although different implementations can be devised, we achieved this by placing a strong weight, w_i_ = 1000, on the dipolar-coupling term when minimizing the Chi square functions, as

6 $$ \chi^{2} = \sum\limits_{i} {w_{i} \frac{{(X_{i,calc} - X_{i,\exp } )^{2} }}{{\sigma_{i,\exp }^{2} }}} $$

This implementation allows keeping the same minimization algorithm. Minimization of the target function was done both by grid search and by the fmin function in numpy, both of which yielding essentially identical results (with the latter one being faster).

All reported error margins on relaxation rates, dipolar couplings and fitted motional parameters were obtained from standard Monte Carlo simulation approaches (Motulsky and Christopoulos 2003).

The F-test analysis that is shown in Figure S6 in the Supporting Information was performed using standard methods that can be found elsewhere, e.g. in (Motulsky and Christopoulos 2003), and are just briefly summarized as follows.

The F-ratio, as shown in Figure S6, was calculated for each residue as:

7 $$ F = \frac{{(\chi_{SMF}^{2} - \chi_{EMF}^{2} )/\chi_{EMF}^{2} }}{{(DF_{SMF} - DF_{EMF} )/DF_{EMF} }} $$

Here, DF_SMF_ and DF_EMF_ refer to the degrees of freedom in the fit of simple and extended model-free, respectively. The degrees of freedom are given by the number of experimental data minus 2 (SMF) or minus 4 (EMF). A probability value was obtained from this F value using a function implemented in the stats module of python.

## Theoretical considerations

**Fig. 1:**
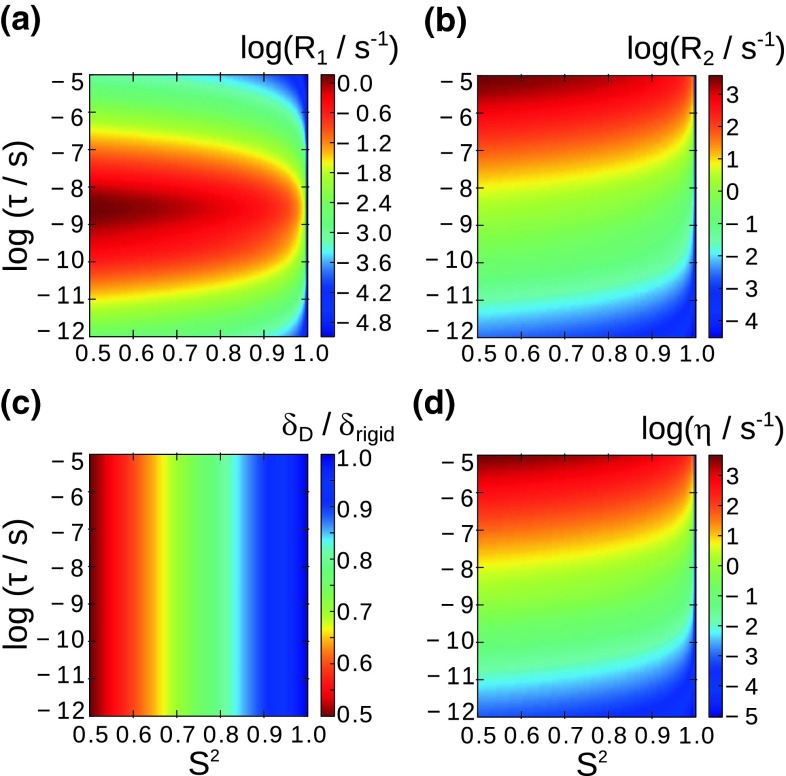
Dependencies of **a** longitudinal ^15^N relaxation rates R_1_, **b** transverse relaxation, R_2_, **c** ^1^H–^15^N dipolar order parameters, given as the ratio of measured and rigid-limit dipolar coupling, and **d** ^1^H–^15^N dipole-^15^N CSA cross-correlated relaxation rate constants on the amplitude and time scale of motion in the framework of the simple model-free description

Figure 1 shows the computed relaxation rate constants for ^15^N longitudinal relaxation R_1_, and transverse relaxation, i.e. R_2_ (that can be obtained at fast spinning from R_1ρ_ measurements (Lewandowski et al. 2011)), and ^1^H-^15^N dipole/^15^N CSA cross-correlated relaxation (Chevelkov and Reif 2008) (in the following denoted briefly as “CCR”), as a function of the amplitude of motion and time scale within the simple model-free (SMF) approach. These relaxation rates are shown for time scales ranging from picoseconds—a time scale often found in solution-state analyses of local backbone fluctuations—to microseconds, where the Redfield theory reaches its limit of validity (Redfield 1957). These plots show that R_1_ relaxation is most sensitive to motion on time scales of nanoseconds, as expected from its dependence on J(ω_N_); both R_2_ and the dipole-CSA CCR are sensitive to motions on time scales exceeding about 1 ns (leading to a measurable relaxation rate of about 1 s^−1^). For completeness, Fig. 1 also shows the information content from dipolar couplings measurements, which directly reflect the motional amplitude, independently of the time scale.

The measurement of a single relaxation rate yields only very limited information, constraining the amplitude and time scale of motion to all combinations of S^2^ and τ falling on a given contour line in Fig. 1a, b, d. Obtaining amplitudes and time scales of motion from relaxation data requires the measurement of several relaxation data. Due to the different dependencies of longitudinal and transverse relaxation rates on motional parameters, it may be possible to derive these parameters from measurement of R_1_ and R_2_/CCR measurements at a single static magnetic field. In addition, one may complement such data with measurements at different field strengths, as these relaxation rates (slightly) depend on the field strength (see Supporting Information in (Schanda et al. 2010)). To investigate how well such an approach would perform in practice, we calculated in-silico relaxation rates for a number of dynamic scenarios, and subjected them to a fit routine, assuming realistic error margins on the rate constants.

**Fig. 2:**
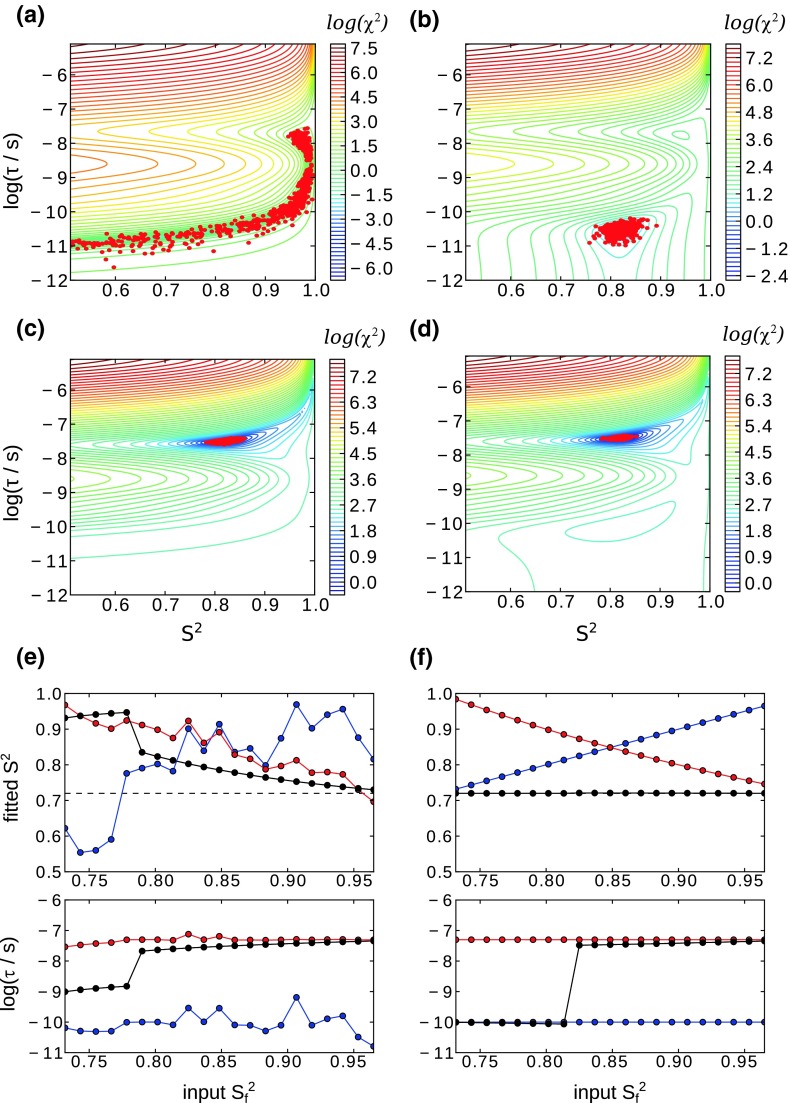
Investigation of the robustness of fitting the amplitude and time scale of motion from different types of data. The *left column* shows fits using relaxation data alone, while the *right column* shows fits of relaxation and dipolar-coupling derived order parameters. In **a**, **b**, a single motion, with order parameter S^2^ = 0.82 and τ = 3.2 × 10^−11^ s was assumed. From these parameters, ^15^N relaxation rate constants (R_1_, R_2_ and η) were back-calculated at a static magnetic field strength of 14.09 T via the model-free approach. In **a**, these three relaxation rates were fitted in the framework of the SMF approach. Shown is the χ^2^ surface of obtained from a grid search. A rather poorly defined minimum extending over a wide range of S^2^ values is found. *Red points* shown the best fits of 2000 Monte Carlo runs, obtained from varying these synthetic relaxation rates within error margins of 0.009 s^−1^ for R_1_, 0.46 s^−1^ for R_2_ and 1.57 s^−1^ for η, which are typical average values found in the present and a previous study (Schanda et al. 2010). The swallow minimum of the target function results in a large error margin on S^2^ in such a Monte Carlo error estimation. In **b** the dipolar order parameter is added to the relaxation data, greatly improving the accuracy and precision of the determined motional parameters. The error margin on the dipolar order parameter S^2^ ((δ_D_/δ_D,rigid_)^2^) was 0.018. The dipolar coupling was treated equally as the relaxation data, as in Eq. 5. In **c**, **d**, the same analysis is performed with S^2^ = 0.82 and τ = 3.2 × 10^−8^ s. In **e**, **f**, the motion is assumed to be according to the EMF model (slow and fast motions), with correlation times of τ_s_ = 5 × 10^−8^ s and τ_f_ = 1 × 10^−10^ s. The total order parameters S^2^ = S_s_^2^ × S_f_^2^ = 0.72 and the S_f_^2^ is varied as shown along the x-axis. Six relaxation rate constants were back-calculated (R_1_ at 11.74, 14.09, 19.96T, R_2_ at 14.09T, η at 14.09 and 19.96T) and fitted in the framework of either the SMF (*black*) or the EMF (*blue*: S_f_^2^, *red*: S_s_^2^), and the resulting order parameters are reported. In **f**, the total order parameter in the fit was fixed to the dipolar-derived value. The *upper panels* in **e**, **f** show the resulting fitted values of S^2^, while the lower panels show the correlation times. In all panels of **e**, **f**, black depicts data from the SMF model, while *blue* and *red* correspond to the fast and slow components of the EMF model, respectively

To this end, we have assumed a N–H bond vector that undergoes motion that is described by one order parameter and one time scale (SMF). Relaxation rates and dipolar couplings were back-calculated for different settings of S^2^ and τ, random noise was added and the data were fit with the SMF formalism. Figure 2a–d show the results of such fits for the case that the motion is in the picosecond range (a, b), or in the nanosecond range (c, d). If only relaxation data are used (panel a), and if the motion is fast then the fit does not provide reliable results, and the order parameter is very poorly defined. Given the insensitivity of transverse relaxation parameters to fast motion (see Fig. 1), this behavior is expected. Interestingly, even the inclusion of relaxation data at multiple fields does not significantly improve the situation, and the uncertainty of the fit remains essentially the same (data not shown). If the motion is in the nanosecond range, the use of relaxation data alone provides reasonable estimates of the motion (Fig. 2c), as transverse relaxation data contain information about motion on this time scale. The situation is generally greatly improved if dipolar coupling data are available (Fig. 2b, d), and in this case both the time scale and the order parameter are correctly obtained, irrespective of the time scale of motion.

It appears unlikely that the backbone exhibits only one single motional mode over the range of time scales that the experimental observables are sensitive to (ps–μs). Therefore, we performed a similar investigation, assuming two distinct motional modes, within the extended model-free approach of Eq. 4 (EMF). As above, various values of amplitudes and time scales of the two motional modes were assumed. The resulting back-calculated relaxation rate constants were fitted with the SMF and EMF approach. Here we assumed that the total order parameter is constant, and the two order parameters, S_f_^2^ and S_s_^2^, are varied. The results of such fits, are shown in Fig. 2e, f. If only relaxation data are used, and the data are fitted with the SMF approach, then the resulting order parameter is always overestimated. This overestimation is particularly pronounced if the underlying motion is predominantly fast, i.e. if S_f_^2^ is low (and, according to our assumption, S_s_^2^ is high). Again, this reflects the fact that relaxation data alone are not capable of correctly picking up fast motion. This mirrors recent studies, where the analysis of relaxation data showed systematically overestimated order parameters (Lewandowski et al. 2011; Mollica et al. 2012).

Fitting the EMF model to relaxation data only essentially fails, as the parameter space is not sufficiently restrained, as was also reported elsewhere (Mollica et al. 2012). Given that in these analyses a total of 6 relaxation data were used, with 3 magnetic field strengths, it appears unlikely that the addition of even more static magnetic field strengths will improve the situation significantly.

The inclusion of dipolar coupling data changes this situation significantly, as shown in Fig. 2f. The order parameter is directly given by the dipolar coupling and therefore, trivially, this value is always correctly retrieved. In the EMF case, the two individual order parameters, S_f_^2^ and S_s_^2^, as well as the two correlation times are all correctly obtained. When these data are fitted within the SMF approach, i.e. an oversimplified model, then necessarily the motion is either fast or slow. Interestingly, the fitted correlation time obtained in the SMF fit is very close to one of the two values assumed (lower panel in Fig. 2f). Whether the SMF fit retrieves a fast motion or a slow motion depends on their relative amplitudes, S_f_^2^ and S_s_^2^, i.e. the fitted τ jumps the fast to the slow regime once the amplitude of the slow motion exceeds a certain level.

These in-silico considerations show that relaxation data alone, even if measured at multiple field strengths, do not provide satisfactory fits, and often lead to systematic errors of order parameters, as sub-nanosecond motion cannot be detected properly with this approach. Only if dipolar couplings are measured, accurate data can be obtained. In the following section we, therefore, investigate how dipolar couplings, which are crucial for obtaining reliable measures of motion, can be measured at high accuracy.

## Measurement of one-bond H–X dipolar couplings from REDOR

A number of recoupling sequences have been proposed for the measurement of heteronuclear dipolar couplings in proteins, in particular TMREV (Helmus et al. 2010; Hohwy et al. 2000), R sequences (Hou et al. 2011, 2013; Levitt 2002; Yang et al. 2009), phase-inverted CP (Chevelkov et al. 2009b; Dvinskikh et al. 2003), DIPSHIFT (Franks et al. 2005; Munowitz et al. 1981) and REDOR (Gullion and Schaefer 1989; Schanda et al. 2010). A detailed description of these pulse sequences and their relative merits and weaknesses is not within the scope of this manuscript. We have recently investigated the robustness of most of these different experimental approaches with respect to experimental artefacts, such as rf field mis-settings and remote spin effects (Schanda et al. 2011b), by extensive numerical simulations. The primary source of systematic experimental errors in most of these approaches are mis-set rf field strengths employed during the recoupling pulse train, as well as the inevitable rf inhomogeneity. Notably, systematic errors on δ_D_ in the range of several percent are easily incurred in many of these recoupling approaches, even if the rf fields are only slightly offset. A notable exception seems to be the case of an approach based on R-sequences, which have been reported to be more robust, at least if samples are center-packed and if three different experiments are measured and fitted simultaneously (Hou et al. 2013).

In the present case of dynamics measurements a systematic error even of only a few percent is a major concern: as the motional amplitude is reflected by (1–S^2^), an error of a few percent on δ_D_, and thus, S (thereby quadratically impacting S^2^) can easily lead to an error of the motional amplitude (1–S^2^), by several tens of percent. In the numerical analysis of different measurement schemes, a time-shifted REDOR approach (Schanda et al. 2010) turned out to be the most robust approach, provided proper calibration of the RF fields. REDOR has the additional advantage that fitting is very robust and straightforward: as the data are obtained in a normalized manner (using a reference experiment), one can fit the data with a single parameter (the dipolar coupling of interest). Most other approaches require fitting signal intensities and line widths (and a zero-frequency component in the dipolar spectrum, that is often left our from the fit in a somewhat arbitrary manner) along with the dipolar coupling. These factors motivated our choice to focus here on the REDOR approach, and investigate experimentally how accurately the obtained order parameters can be measured, and how mis-settings of ^1^H and ^15^N π pulse power impact the apparent measured dipolar coupling.

**Fig. 3:**
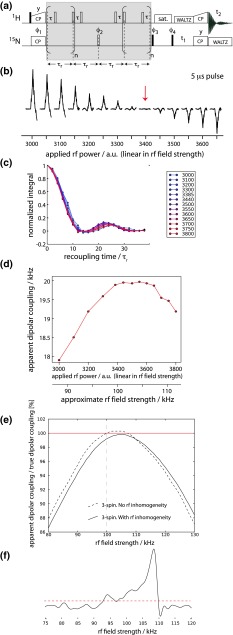
Measurement of ^1^H-^15^N dipolar couplings with REDOR. **a** Pulse sequence used in this study. **b** Calibration of ^1^H π pulses, achieved by setting the initial ^1^H excitation pulse in **a** to 5 μs, and varying the rf power. The *grey shaded box* in **a** was omitted for this experiment. A π rotation is achieved at the rf power level where the zero-crossing is observed. **c** REDOR oscillation curves measured in a 1D manner on microcrystalline ubiquitin, using rf power levels corresponding to the ones shown in **b**. **d** Dipolar coupling values, obtained from fitting the data shown in **c**. **e** Numerical simulations of the REDOR experiment with different ^1^H rf power levels (5 μs duration pulse). Shown are simulations of 3-spin H-H-N systems, where the remote H was set at a distance of 2.6 Å to the proton and 4.1 Å to the nitrogen spin, corresponding to dipolar tensor anisotropies of and 13,668 and 353 Hz, respectively, according to the definition of the dipolar coupling tensor in (Schanda et al. 2010). The Euler angles describing the spin system are as follows (listed as α, β, γ, in degrees): D(N–H^1^): 0, 0, 0; CSA(N): 0, 20, 0; D(N–H^2^): 0, 70, 0; D(H^1^–H^2^): 0, 22,0; CSA(H^1^, σ_zz_ = 1200 Hz): 0, 29, 0; CSA(H^1^, σ_zz_ = 900 Hz): 0, −30, 0. The direct N-H coupling was set to 20.4 kHz. Chemical shift offsets were assumed on the three spins as 100 Hz (N), 600 Hz (H^1^) and 1,200 Hz (H^2^). Note that the chemical shift offsets and CSA tensors have only very small effects (Schanda et al. 2011b). The dashed line assumes a single ^1^H rf field strength, while the *solid line* assumes a distribution of ^1^H rf fields. This distribution was assumed to correspond to the experimentally observed one, shown in **f**, by adding up a range of simulations with different ^1^H rf field (step size 1 kHz) according to the nutation spectrum of **f**. The threshold for this summation is indicated as a *dashed line*

Figure 3 shows the pulse sequence that we employed here for measuring ^1^H–^15^N dipolar couplings in deuterated proteins, and some experimental data obtained on a microcrystalline sample of u-^2^H^15^N-labelled ubiquitin, reprotonated at 50 % of the amide sites and undergoing MAS at ν_r_ = 37.037 kHz (τ_r_ = 27 μs). Akin to a previously proposed experiment (Schanda et al. 2010), the central REDOR sequence element in Fig. 3a features ^1^H π pulses that are shifted away from the middle of the rotor period. This allows scaling down the effective dipolar evolution and thereby sampling the recoupling curve more completely on the sampling grid that is dictated by the rotor period (Gullion and Schaefer 1989). Provided that the ^1^H spin network is diluted (deuterated sample, as used here), the main source of artifacts is mis-setting of rf fields (Schanda et al. 2011b). It is therefore instructive to inspect the effect of different calibrations of the π pulses on the apparent REDOR recoupling.

Calibrating rf fields to high precision is not trivial, and calibrations obtained from different methods might not match. For example, we find that the zero-crossing found when replacing a π/2 pulse by a π pulse does not necessarily match the calibration obtained from nutation experiments, where the pulse duration is varied over a large range, or calibration via rotary resonance conditions (data not shown). The possible source of error in all these rf calibrations are finite pulse rise times, amplifier droops or phase transients. This, of course, complicates the situation in many recoupling techniques, where a train of (phase-switched) back-to-back pulses is applied. In the case of the REDOR experiment, the situation is more easily tractable, as it consists of a train of well-separated individual π pulses; phase transients and amplifier droops should thus not be a major concern, and calibration of the π pulse by searching a zero-crossing, thus, appears as the most appropriate way of calibration.

Figure 3b shows a calibration of the ^1^H π pulse, obtained by replacing the initial excitation pulse (Fig. 3a) by a 5 μs pulse, and varying the rf power in the vicinity of the expected 100 kHz (i.e. searching for a zero-crossing). Figure 3c shows REDOR curves, obtained for the different ^1^H π pulse power levels during the recoupling, which correspond to the values shown in Fig. 3b. These curves were obtained by integration over the entire amide spectrum. The resulting fitted dipolar couplings are shown in Fig. 3d, assuming that the REDOR curves can be represented by a single value of δ_D_.

These data show that the obtained dipolar coupling depends only slightly on the ^1^H rf field setting, as long as the rf field is close to the value found for the zero-crossing. The apparent dipolar coupling has a maximum for an rf field setting slightly higher than the calibrated value from the zero-crossing (Fig. 3b). The rf field strength that corresponds to the nominally correct value of 100 kHz (Fig. 3b) leads to an apparent dipolar coupling slightly below the maximum value (Fig. 3d).

In order to understand this behavior, we have performed numerical simulations, shown in Fig. 3e. The dashed line shows the apparent dipolar coupling, obtained from simulating a three-spin N–H–H system, subjected to REDOR recoupling ^1^H pulses of constant duration (5 μs), but different rf field strength. In agreement with the experimental data, we find that the obtained dipolar coupling slightly depends on the rf field setting, and that the maximum dipolar coupling is seen at an rf field strength slightly above the correct rf field.

In a realistic setting, inhomogeneity of the rf fields across the sample is inevitable. From the experimental data and simulations shown above, it is clear that such a distribution of rf field results in a distribution of REDOR oscillation frequencies over the sample volume. In order to account for this effect, we have experimentally measured the shape of the rf field distribution in the 1.6 mm Agilent fast-MAS probe used here, by performing a nutation experiment. The ^1^H nutation spectrum, obtained from Fourier transformation of a series of 1D spectra with excitation pulses of variable length, shown in the Fig. 3f, reveals a distribution of rf fields over more than 5 kHz, distributed in a non-symmetric manner, i.e. a broader distribution towards lower rf fields, a situation typically found in solenoid coils. The rf power that was used in this experiment is identical to the one for which we found a 5 μs-long π pulse (i.e. a nominal 100 kHz pulse, Fig. 3b). Interestingly, the peak of this observed distribution is above 105 kHz and, thus, well above the field found from the zero-crossing of a single 5 μs pulse (Fig. 3b). We ascribe this finding to pulse rise time effects: when a single 5 μs pulse is applied, the finite pulse rise time results in a reduced flip angle of the spins relative to a perfect rectangular pulse; in the nutation experiment, where the pulse duration is arrayed (at the same power level for which the 5 μs pulse resulted in a π nutation), and pulses over the course of the nutation series go up to durations much longer than 5 μs, these rise time effects have a smaller effect than in the situation where a short pulse is applied. Thus, at the same power level, the rf field strength appears higher than in the single π pulse case.

In order to account for the effect of such rf field distributions, we have explicitly simulated REDOR curves for the above three-spin system at various rf field strengths. Different REDOR curves were then added up with weighting factors according to a profile that matches the breadth and shape of the experimentally observed rf inhomogeneity profile of Fig. 3f. However, the center of mass of the distribution taken for these summations was shifted, such that we can investigate rf mis-setting with simultaneous rf field distribution. The solid line in Fig. 3e shows the fitted values of δ_D_ that are obtained when fitting these simulated curves against perfect two-spin REDOR simulations. We find that the shape of the profile of obtained δ_D_ as a function of the rf field setting is similar to the one that neglects the rf field inhomogeneity (dashed line). However, the obtained δ_D_ are generally lower; this is expected, as the rf field inhomogeneity leads to a situation where parts of the sample are subject to lower rf fields, and thus slower apparent REDOR oscillations.

The effect of this reduction of the apparent dipolar coupling is sizeable, and has to be taken into account when bias-free data should be obtained. This can be done upon data analysis either by fitting experimental data explicitly against simulations that take into account the rf field distribution, or by determining the factor by which the apparent dipolar couplings are reduced—using data as shown in Fig. 3e. While these two approaches are, in priciple, equivalent, the latter is computationally much less costly: it consists of fitting experimental data using a grid of simulations based on standard simulations (that neglect the rf distribution), and applying a correction factor *a posteriori*. In this work we apply this approach. From the simulations in Fig. 3e we find a correction factor of 1.1 % on the values of δ_D_, by which the fitted couplings should be scaled up. This is in good agreement with the factor by which non-scaled dipolar-coupling order parameters (i.e. not corrected for rf inhomogeneity) and solution-state relaxation order parameters differ, which is 1.5 % (see Fig. 5 below). We note that in this analysis we have neglected the possibility that variations of the ^1^H and/or ^15^N CSA tensors may also contribute to some of the offset. As these tensors vary from site to site, no global scaling factor could correct for this effect. For R-type sequences, it has been shown that the ^1^H CSA tensor has an impact on the accuracy of measured heteronuclear dipolar couplings (Hou et al. 2013). For the case of REDOR, previous analyses (Schanda et al. 2011b), as well as investigations shown in Figure S9, show that the systematic errors that CSAs might induce are very small, below 0.5 %, and we thus disregard CSA effects, and identify the rf field setting (and inhomogeneity) as the main point to consider. This is also corroborated by the close match between the scaling factor between REDOR- and solution-state order parameters, and the correction factor we identify from rf inhomogeneity, noted above (1.5 vs. 1.1 %).

Finally, we also investigate whether the behavior shown in Fig. 3 also holds if lower ^1^H rf fields are used. As shown in Figure S10, the behavior found in Fig. 3e is also found if 8 μs pulses are used, instead of 5 μs.

**Fig. 4:**
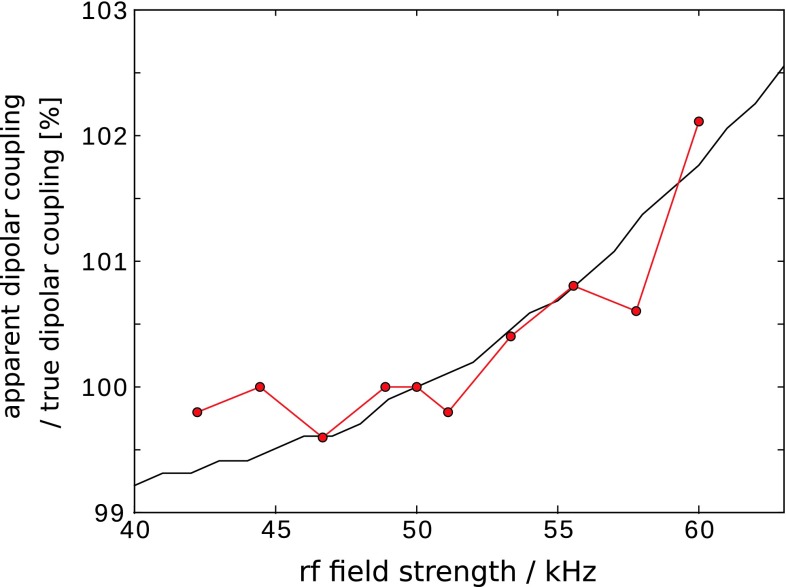
Dependence of the apparent dipolar coupling on the rf field strength of the central ^15^N π pulse in the REDOR experiment of Fig. 3a. The experimental data (*red*) were obtained from 1D REDOR curves in an analogous manner as the data shown in Fig. 3c,d. Different points reflect different rf power level settings. Experimentally, the ^15^N π pulse was calibrated by setting the pulse with phase Φ3 (Fig. 3a) to 10 μs, and searching the rf power that results in zero intensity, analogous to the procedure in **b**. The point at 50 kHz was set according to this calibration. The black solid curve shows simulated data. REDOR experiments were simulated by assuming a H-N dipolar coupling of 20.4 kHz, and perfect 100 kHz (5 μs) ^1^H π pulses and ^15^N π pulses of 10 μs duration and variable field strength. Remote protons and rf field distribution were ignored. The simulations were fitted against ideal two-spin simulations, and the resulting dipolar coupling is reported (relative to the nominal 20.4 kHz value). The red curve was set in the vertical axis such that 100 % is at an rf field of 50 kHz. The *black curve* is normalized to the nominal input value of δ_D_ = 20.4 kHz

We have also investigated the sensitivity of the obtained dipolar couplings to mis-settings of the ^15^N π pulse. Figure 4 demonstrates both experimentally and through simulations that the apparent δ_D_ is much less sensitive to the ^15^N rf field than it is the case for the ^1^H field. Interestingly also, there is not a maximum of δ_D_ for a given rf field strength; thus, rf field inhomogeneities also tend to cancel their relative effects (data not shown). Based on these findings, we carefully calibrate the ^15^N π pulse, and neglect ^15^N rf field mis-settings and inhomogeneities in all analyses.

Finally, we have also considered two different ways of performing the XY-8 phase cycling of the ^1^H π pulses. One possibility is to cycle all pulses according to the XY-8 scheme, from the first pulse to the last pulse, irrespective whether the pulse is applied in the first or second half of the recoupling block. Alternatively, one can also keep the phases symmetrical with respect to the center of the recoupling block, i.e. increment the phases in the first half, and decrement the phases in the second half, as done before (Schanda et al. 2010). Although the differences are rather subtle, we find it preferable to chose the second approach; in the first one we find that the REDOR curves have slightly higher oscillation amplitudes, and the match with simulated recoupling traces is slightly less good (see Figure S1 in the Supporting Information).

### Dipolar order parameters in ubiquitin

**Fig. 5:**
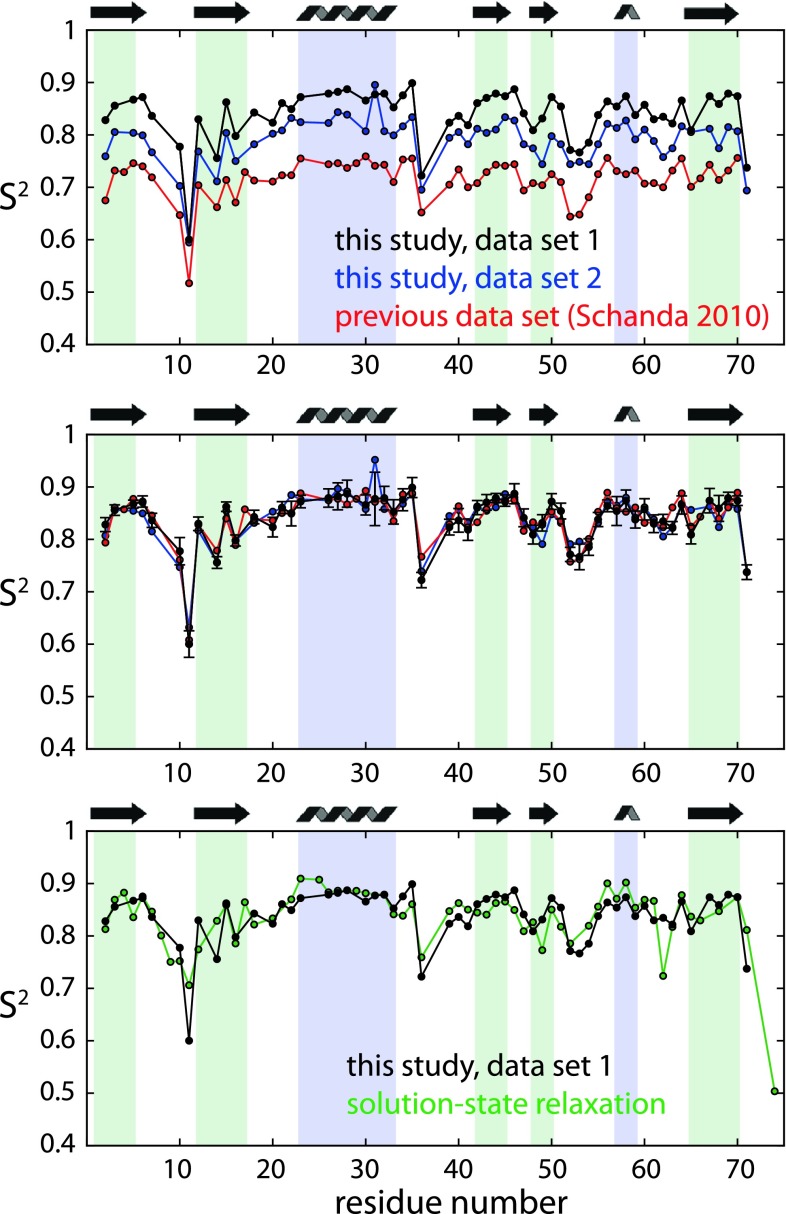
^1^H–^15^N dipolar-coupling derived order parameter in ubiquitin, obtained as S^2^ = (δ_D,exp_/δ_D,rigid_)^2^. Plots of S^2^ are preferred rather than S or δ_D,exp_, as possible offsets and differences are accentuated in such an S^2^ plot. **a** Measured dipolar-coupling derived S^2^ obtained in this study, with the pulse sequence in Fig. 3a, accurate ^1^H π pulse calibration as described in Fig. 3, and correction for the ^1^H rf inhomogeneity are shown in *black*. The data in *red* are the data previously published (Schanda et al. 2010), and data shown in *blue* were data obtained in this study, with a different phase cycling of the ^1^H π pulses (see Figure S1 in the Supporting Information), and somewhat less accurate ^1^H pulse calibration. In **b** the latter two data sets are scaled with one global scaling factor as to reduce the offset to the black data set. The scaling factor that was applied to the values of S shown in the red data set was 1.084, and the factor used for scaling the S values of the blue data set was 1.031. The good reproducibility of the data is evident. Note that the data set shown in *red* is, itself, already an average over three independent measurements, which themselves show high reproducibility of the *S* ^2^ profile (Schanda et al. 2011b). **c** Comparison with solution-state order parameters (Lienin et al. 1998), which were re-interpreted using a ^15^N CSA of Δσ = 170 ppm (data courtesy of R. Brüschweiler). Error bars are omitted for the sake of clarity. A correlation plot of the data in **c** is shown in the Supporting Information (Figure S4)

Figure 5 shows experimental dipolar coupling data obtained on microcrystalline ubiquitin. Representative REDOR curves for individual residues are shown in Figure S1. The black data set was obtained taking into account the ^1^H rf inhomogeneity. Figure 5a, b show, in addition to the data obtained with the procedure outlined above, a data set obtained in a previous study (Schanda et al. 2010), as well as data obtained in the present study with a different implementation of the XY8 phase cycle mentioned in the previous paragraph (see Figure S1). In these latter two data sets, calibration was performed with a somewhat lower degree of accuracy, and the rf inhomogeneity was ignored. In Fig. 5a these two data sets are shown without any scaling, while in Fig. 5b a global scaling parameter has been applied to minimize the offset to the black data set, which is the one described above (with rf field inhomogeneity correction and very accurate pulse calibration). Clearly, these two data sets are systematically lower than the data set that was obtained from the rf calibration and rf inhomogeneity treatment explained above. An underestimation in the other data sets is expected, as any miscalibration and rf inhomogeneity leads to underestimated dipolar couplings (see Fig. 3). It is interesting to note, however, that if the data sets are scaled by one global scaling factor, as shown in Fig. 5b, the agreement is excellent. This shows that the method yields highly reproducible results for the order parameter profile, even though the data were collected on different samples, different probes and different spectrometers.

Notably, the scaling factor that needs to be applied to the previously published data set (Schanda et al. 2010) in order to match the new data set (shown in black in Fig. 5) is rather large (1.084 on S). This large scaling factor cannot be explained by rf inhomogeneities alone, at least not if they are in the same order of magnitude as the rf inhomogeneity found in the probe used here. Although it might be that the probe used in the previous study has a larger inhomogeneity, we rather speculate that the rf calibration in the previous study was not accurate (possibly it was done from a nutation rather than a π pulse optimization), which might explain the offset. Another finding points in the direction of wrong rf calibration: in the previous data set the experiment was measured three times, using two different ^1^H rf fields (100, 125 kHz) and two different delays τ) for one of the two rf fields. While the data sets using the same rf field strength (100 kHz) resulted in very similar values, the data set at 125 kHz ^1^H rf field is slightly offset (although within error bars) (Schanda et al. 2011b). This rather suggests that the rf calibration was not perfect.

Figure 5c shows a comparison of the present order parameters with values derived from solution-state measurements (Lienin et al. 1998). This comparison reveals that, overall, the solid-state data are in very good agreement with the solution-state data, confirming previous findings that sub-microsecond protein dynamics is very similar in solution and crystals (Agarwal et al. 2008; Chevelkov et al. 2010).

The above analysis allows establishing guidelines for obtaining dipolar-coupling-derived order parameters with high accuracy in deuterated samples. (1) REDOR recoupling pulses should best be calibrated by directly searching the π pulse power, not via nutation experiments, as this best reflects the actual situation in the REDOR pulse train. (2) Once correct pulse calibration is used, RF field inhomogeneities slightly alter the outcome of the experiment, and these inhomogeneities should be taken into account by explicity measuring the rf profile of the probe. Simulations can establish the scaling factor by which raw fitted data should be scaled. We estimate that with these careful calibrations and corrections, the systematic error of the obtained dipolar couplings can be below 1% at most, as suggested also by the close correspondence of solution- and solid-state order parameters.

### Transverse relaxation rates from R_1ρ_ measurements at ~40 kHz MAS

With the aim of obtaining a data set that is as comprehensive as possible, we furthermore measured ^15^N R_1ρ_ relaxation data. Transverse relaxation data are inherently difficult to measure, due to the presence of coherent mechanisms of coherence loss, such as dipolar dephasing. A recent study indicated that fast MAS (about 40 kHz or more) can avoid these problems and provide access to the pure R_1ρ_ relaxation part of the coherence decay even in the dense network of a protonated protein and in the absence of proton decoupling (Lewandowski et al. 2011). Another study proposed the use of highly deuterated (20% amide-protonated) samples to obtain clean R_1ρ_ rates (Krushelnitsky et al. 2010). Here we use both a highly deuterated sample and fast MAS (39.5 kHz) to measure ^15^N R_1ρ_ rates at an rf field strength of 15 kHz. There is strong evidence that the obtained rate constants truly reflect dynamics, because (1) back-calculated R_1ρ_ rates obtained from a model-free fit of 5 relaxation data sets and the dipolar coupling measurements are in good agreement with the experimentally obtained values of R_1ρ_ (see Figure S2), and (2) R_1ρ_ rates are independent of the rf field strength in the range explored (5–15 kHz; data not shown).

### Fitting backbone motion from multiple data sets

In the following, we explore how the available relaxation data and dipolar couplings can be interpreted in a physical model of backbone motion. Altogether, we use up to 7 data sets (in cases of resonance overlap, for some residues less data may be available).

- ^15^N R_1_ at field strengths corresponding to 500, 600 and 850 MHz ^1^H Larmor frequency (Schanda et al. 2010)
- ^1^H-^15^N dipole-^15^N CSA cross-correlated relaxation at 600 and 850 MHz (Schanda et al. 2010)
- ^15^N R_1ρ_ at 600 MHz (this study, values reported in the Supporting information)
- ^1^H–^15^N dipole couplings (this study, values reported in the Supporting information)

(All relaxation data are shown in Figure S3.) As in the theoretical section above, we use either the one-time scale simple model-free (Lipari and Szabo 1982b) or the two-time scale extended model-free approach (Clore et al. 1990).

**Fig. 6:**
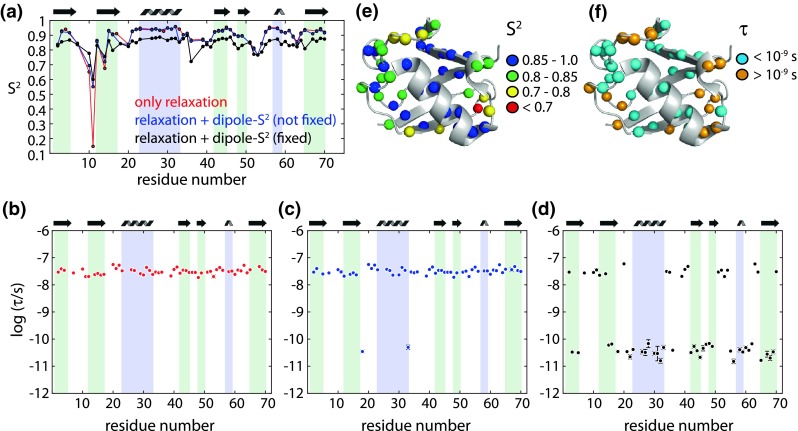
SMF fit of relaxation and dipolar coupling data in ubiquitin. **a** Results from fitting only relaxation data (up to 3× R_1_, 2× η and 1× R_1ρ_ per residue) are shown in *red*. Inclusion of dipolar coupling data results in the *blue* data set. In this data set, the order parameter was not fixed to the dipolar order parameter, but the dipole coupling was included in the fitting of S^2^ and τ in the same manner as the relaxation data, as shown in Eq. (3). In the black data set, the order parameter was fixed to the dipolar-coupling derived value. **b**–**d** show the fitted time scales for the three scenarios, using the same color code. The fitted order parameters and time scales from the fit where S^2^ was fixed to the dipolar-coupling derived value (*black curves*) are plotted on the structure in **e**, **f**. In the fits that included dipolar coupling data, a minimum of 3 data points was required for a residue to be considered; in the fit with relaxation data only, a minimum of 4 data points was required

Figure 6 shows fit results for the SMF approach, using three different implementations. In one case, only the 6 relaxation data sets were used; S^2^ are reported as red curve in (a) and the corresponding τ shown in panel (b). In another implementation, dipolar couplings were added to the fit, but the fitted S^2^ was not imposed to match the dipolar-coupling derived one; rather, all relaxation and dipolar-coupling data were equally used for a χ^2^ minimization, according to Eq. 5 [S^2^ shown as blue data set in panel (a), τ in panel (c)]. Finally, a similar fit was performed, but this time the order parameter was fixed to the dipolar-coupling derived value [black curve and panel (d)].

If only relaxation data are used, the obtained order parameters are systematically overestimated, as compared to the dipolar order parameter. Furthermore, the time scale of motion is in the nanosecond range for all residues. This overestimation of S^2^ by relaxation data, as well as the finding of nanosecond motion only is in agreement with the above in-silico data (Fig. 2). Although there is no physical foundation for such an approach, one might be tempted to search for a scaling factor, that would bring the relaxation-derived S^2^ to the level of the dipolar ones. Mollica et al. have shown that for their data set on GB1, that a scaling factor of 0.96 results in reasonable agreement with MD-derived order parameters. We have applied a similar procedure, and find that a scaling factor of 0.967 results in an overall similar level of order parameters, while a factor of 0.93 leads to best match for secondary structure elements. However, this apparent similarity merely reflects the fact that the backbone mobility tends to have a similar level throughout the protein, so it is always possible to find a scaling factor that makes these levels look similar. (A correlation plot of the data in Fig. 6a is shown in Figure S4). Such a scaling approach does not have physical foundation and is not expected to provide physically meaningful data.

Interestingly, if dipolar couplings are added to the SMF fit, but treated in the same manner as relaxation data (i.e. S^2^ is not fixed to its dipolar-coupling derived value), the situation does not greatly improve, and a similar level of S^2^ is found as if only relaxation data are used (blue data set in Fig. 6). This reflects the fact that the larger number of relaxation data outweighs the contribution from the dipolar data in the target χ^2^ function. In contrast, if S^2^ is fixed to the REDOR-derived value, which are in close agreement with solution-state S^2^ (Fig. 5), an interesting pattern of correlation times is observed, where values of τ fall either in the fast or the slow regime (Fig. 6d). This clustering basically corresponds to τ values falling either above or below the regime where the ^15^N R_1_ is maximum (see Fig. 1). Interestingly, residues for which we observe a slow motional time scale correspond almost exclusively to loop regions, while the residues for which the SMF fit shows a picosecond motion are mostly located in secondary structure elements. This observation is in line with the fact that loop motions are generally the result of concerted motion of several residues, which is a more rare event than localized motion. Of note, the fit that used only relaxation data did not detect this feature, and all the residues showed only motions on long time scales (tens of nanoseconds). Similar findings of exclusive nanosecond motion were reported also in previous relaxation-based analyses (Lewandowski et al. 2010b). Based on the in-silico analyses, and on the comparison with the fit including dipolar coupling data, we conclude that this detection of exclusively slow motion for all residues is essentially an artifact arising from fitting relaxation data only.

**Fig. 7:**
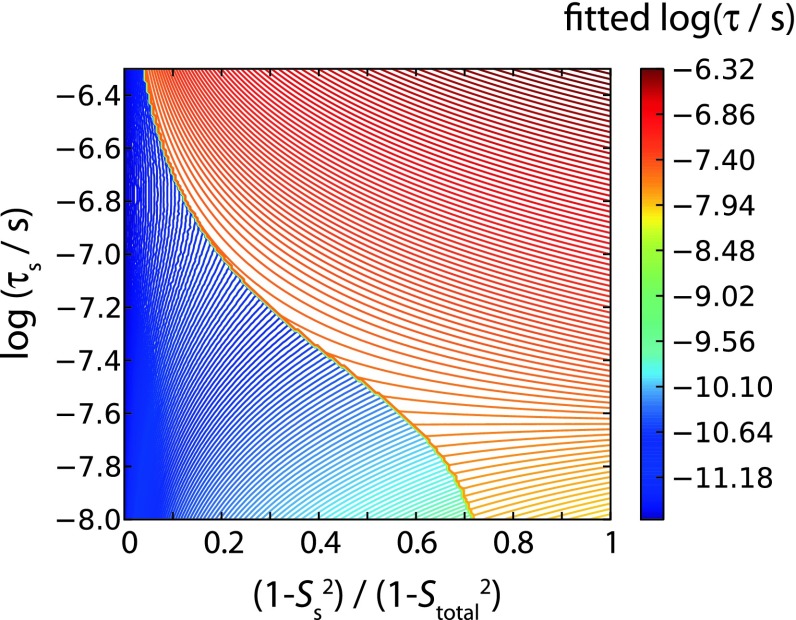
Investigation of the outcome of SMF fits when applied to a two-time scale motion. Shown is the fitted correlation time of motion in an SMF fit, applied to in-silico relaxation (R_1_, R_1ρ_ at 600 MHz) and dipolar-coupling data, calculated from an EMF model. The slow time scale, τ_s_, used in the EMF model, is shown along the vertical axis, while the amplitude of the slow motion (1–S_s_^2^), relative to the the total amplitude of motion (1–S_total_^2^), where S_total_^2^ = S_f_^2^ × S_s_^2^, is shown along the horizontal axis. The correlation time of fast motion, τ_f_, was assumed as 2 × 10^−12^s. Plots for other values of τ_f_ are shown in Figure S5

The SMF approach is tempting for its small number of fit parameters, which makes it applicable even if only one field strength is available. However, the assumption that backbone motion over 6 orders of magnitude in time can be described as a single process appears too simplistic. From a physical point of view, it seems more realistic that for those residues that exhibit slow motion in Fig. 6d, the slow motion dominates, rather than being exclusive. We tried to investigate how the simultaneous presence of slow and fast motion would impact a SMF fit procedure. To this end, we performed an analysis extending on the above theoretical considerations of Fig. 2f. We assumed that the actual motion can be described with the (somewhat more realistic) EMF model; we systematically varied all the parameters of the model (S_f_^2^, S_s_^2^, τ_f_, τ_s_), back-calculated relaxation and dipolar-coupling data from these parameters, and then fitted them through an SMF approach. A representative plot of these data is shown in Fig. 7. Whether the SMF-derived correlation time falls into the slow or fast regime not only depends on the relative amplitudes of slow and fast motion, but also on the correlation times. For example, in the case that the time scale of the slow motion is long (hundreds of nanoseconds) the SMF fit would find a slow motion even if the amplitude of that motion is much smaller than the amplitude of the simultaneously present fast motion (see Fig. 7). This is expected, as large transverse relaxation rate constants can result even from very low-amplitude motions, as long as the time scale is long enough. We also note that the plot shown in Fig. 7 does not depend on the total amplitude of motion, but only on the fast-motion correlation time, τ_f_ (Figure S5).

We conclude from this analysis that our finding of slow motion for a number of loop residues in ubiquitin (Fig. 6d) does not mean that there is no fast motion in the concerned regions, nor does it necessarily mean that the amplitude of slow motion is larger than the amplitude of fast motion, as both the time scale and the amplitude are decisive for whether slow motion is detected in the SMF fit.

**Fig. 8:**
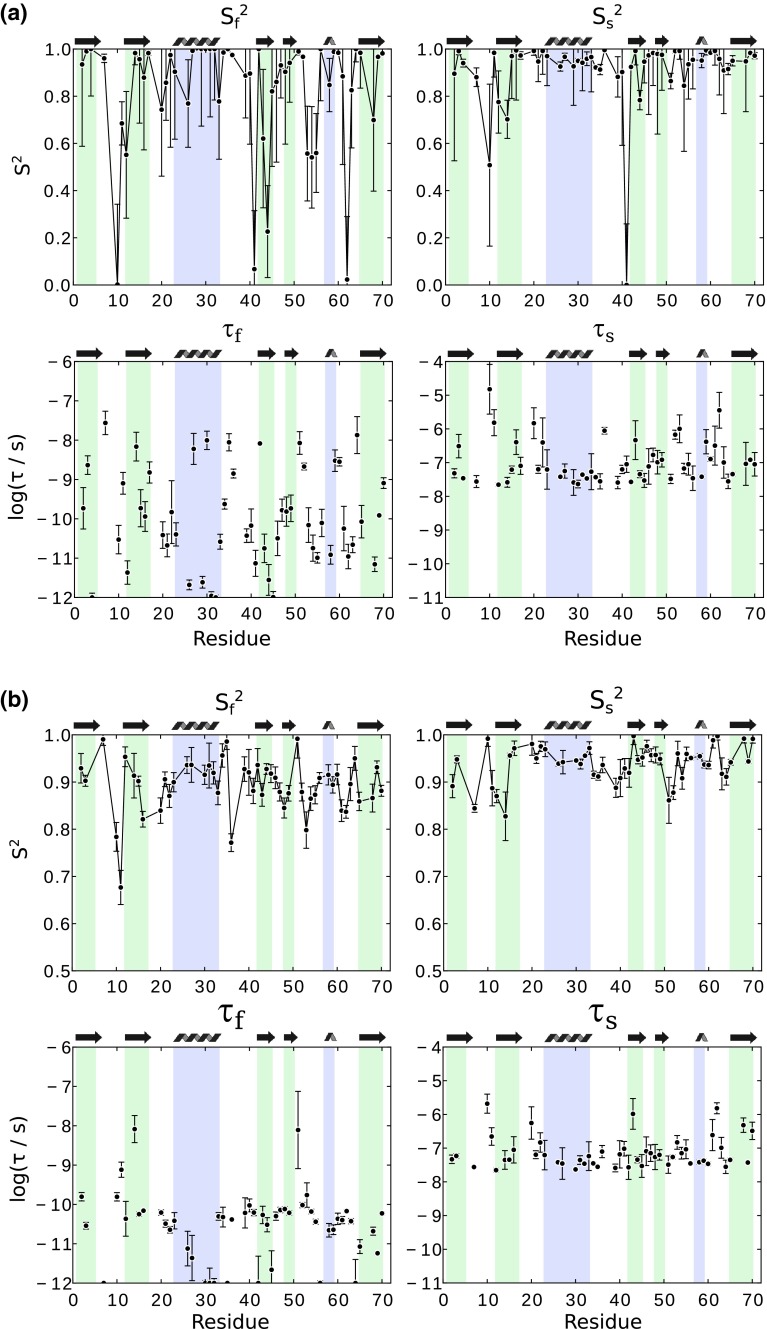
EMF fit of relaxation data only **a**, and with relaxation data and dipolar couplings **b**. In **b**, the overall order parameter S_s_^2^ × S_f_^2^ was fixed to the REDOR-derived value. Only residues for which at least 4 data points are available were considered

We also fitted the more complex EMF model with two motional time scales to our data (i.e. 4 fit parameters). If only relaxation data are used, even if measured at multiple fields (6 data sets in our case), the fit results in an underdetermined parameter space, i.e. very large error bars, and physically rather unrealistic fit parameters, such as high order parameters (Fig. 8a).

Figure 8b shows results of an EMF fit to relaxation and dipolar-coupling data. A number of physically intuitive patterns emerge from this fit. Slow-motion order parameters tend to be lowest in loop regions, while some secondary structure elements have S_s_^2^ close to unity; the lowest fast-motion order parameters are found in loop regions, similar to solution-state analyses. The time scale of fast motion is in the range of tens to hundreds of picoseconds, while slow-motion correlation times are in the range of tens of nanoseconds for most residues, while for some residues we find values up to about one microsecond. The EMF fit also shows some features that are physically less intuitive. For example, residue 10, located in a loop and exhibiting enhanced transverse relaxation, has a slow-motion close to unity, but a very long correlation time. It’s neighbor, residue 11, has a significantly lower S_s_^2^, and a correlation time that is one order of magnitude shorter.

A statistical analysis of the two fit models, SMF and EMF, using F-test reveals that the EMF model is the accepted model for a 31 out of the 46 residues (Figure S6). In contrast, however, a Akaike Information Criterion test rejects the EMF model for all residues (data not shown). To get further information about the robustness of the EMF fit, we systematically eliminated individual data sets from the fit. The results of these fits (shown in Figure S7), reveal that many of the features are retained if data sets are eliminated, e.g. the amplitude of slow motion is generally smaller than the fast-motion amplitude. However, when seen at a per-residue level, the relative amount of fast vs slow motion, as well as the correlation times, vary in some cases substantially when data sets are eliminated, even for residues that are fitted significantly better with EMF (according to an F-test). As expected, the SMF model is much more robust to elimination of individual data sets, and the fitted correlation time is hardly sensitive, at least as long as both longitudinal and transverse relaxation data are available (Figure S7).

Getting a large set of relaxation data, in particular measurements at multiple field strengths, is often impracticable. Practical problems with multiple-field measurements include the availability of multiple NMR magnets, and fast-MAS probes at the different magnets (as the relaxation rates are best measured at fast spinning), and possibly the need for preparing multiple rotors for the different probes, which may cause problems of comparability of different preparations. In addition, the temperatures in different measurements on different probes may not be exactly identical. Therefore, we investigated the information that can be obtained from fitting data collected at only one magnetic field strength, i.e. using only ^15^N R_1_, ^15^N R_1ρ_ and ^1^H-^15^N dipole couplings. We left out the dipolar-CSA CCR data, as their information content is similar to the on of R_1ρ_, while in our hands the latter can be measured with higher precision.

**Fig. 9:**
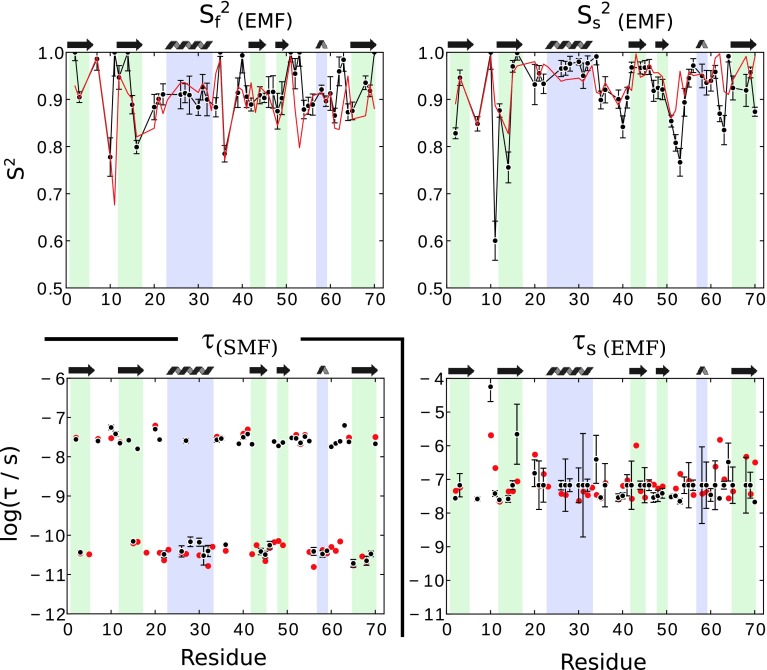
Model-free fits from data obtained at a single magnetic field strength (14.09 T), using dipolar-coupling derived S^2^, R_1_ and R_1ρ_. Shown are fitted parameters for the SMF and EMF cases. In both cases, the overall order parameter, i.e. the S^2^ in SMF, or S_s_^2^ × S_f_^2^ in EMF, was fixed to the dipolar S^2^. In the SMF case, only the time scale is shown, as S^2^ is identical to the data shown in Fig. 5. In the EMF case, the time scale of fast motion, τ_f_, was fixed to 80 ps, as the number of fitted parameters would otherwise exceed the number of observables. Fits using other assumed τ_f_ are shown in the Supporting Information Figure S8. For comparison, the fit parameters obtained from a fit of all available experimental data (up to 7) are shown in red. In these fits, for all residues three data sets were used (R_1_, R_2_, S^2^)

Figure 9 shows results from such fits using data obtained at a single B_0_ field (14.1T). In the case of SMF, the obtained fitted correlation times agree remarkably well with the ones obtained from the full data set that comprises 6 relaxation rates (instead of 2 used here). Note that in this fit the relaxation data serve only to constrain the correlation time, as the order parameter is defined by the dipolar-coupling measurement.

We also investigated EMF fits from a limited data set. Obviously, fitting four parameters from three experimental data sets is impossible. However, our finding of rather uniform values of τ_f_ (see Fig. 8b) prompted us to set τ_f_ to a fixed value for all residues. Figure 9 shows EMF fit results for the case of τ_f_ = 80 ps. Despite the very limited data set, the fitted parameters are in relatively good agreement with the fit that uses the entire data set. However, the choice of the τ_f_ value has a clear impact on these fits (Figure S8), such that such an approach must be interpreted with some care.

## Comparison of order parameters with solution-state data

**Fig. 10:**
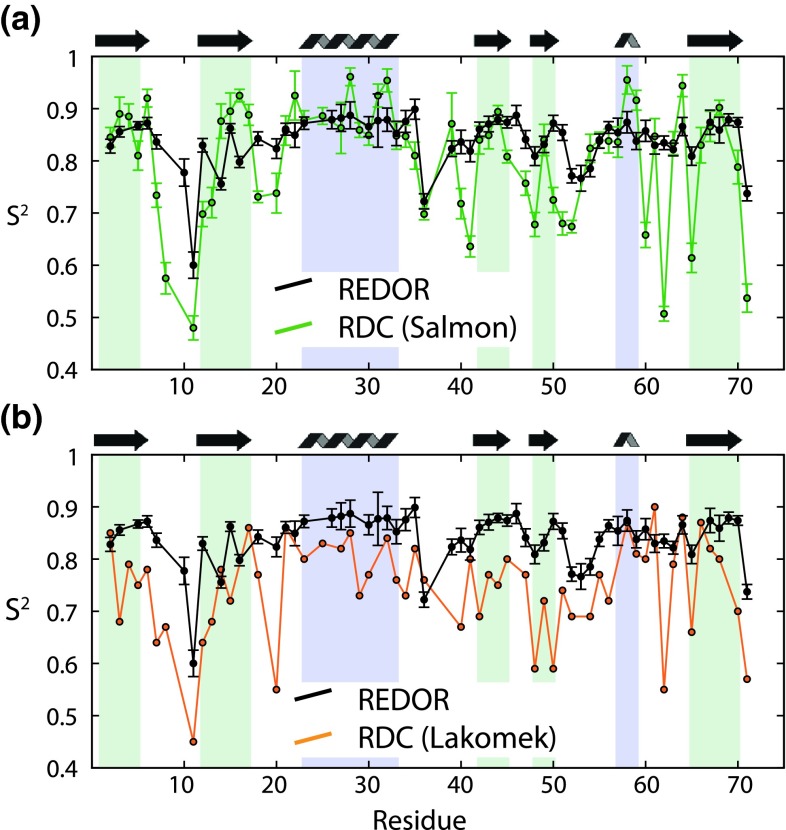
Comparison of REDOR-derived order parameters with RDC-derived S^2^ values in solution, using two different approaches, according to (Salmon et al. 2009) (**a**) and (Lakomek et al. 2008) (**b**)

We have compared above the dynamics on time scales of picoseconds to a few microseconds, as seen by REDOR, with solution-state relaxation data, which are sensitive to a smaller time window, reaching from picoseconds up to a few nanoseconds only. In recent years, a number of studies have addressed protein dynamics in solution from residual dipolar couplings (RDCs). RDCs are sensitive to motion on time scales from ps to ms, and thus overcome the limitation of solution-state relaxation measurements. Due to difficulties to disentangle the amount of alignment in anisotropic solution, the structural component to the RDC, and the amount of dynamics, RDC analyses are challenging. Solid-state dipolar couplings might provide complementary insight, as they are only sensitive to local motion, but not to the structure. It is, thus, interesting to compare our present dipolar-coupling data to order parameters from solution-state RDC analyses. Of course, one does not necessarily expect perfect agreement between these data sets, because dynamics may be impacted by the crystalline environment (Tollinger et al. 2012). Figure 10 shows the comparison of REDOR-derived S^2^ with S^2^ derived from an extensive set of RDC data, analyzed with two different approaches (Lakomek et al. 2008; Salmon et al. 2009). Overall, the amplitude of motion seen in our REDOR data appears to match better the data set shown in (a) (Salmon et al. 2009) than the one in (b) (Lakomek et al. 2008). In both cases, a number of notable differences can be seen between solution-state RDC order parameters, and REDOR order parameters. Notably, the RDC-derived order parameters have much more site-to-site variation. This may appear surprising, as the solution-state relaxation-derived order parameters agree much better with the solid-state REDOR order parameters (Fig. 5c). For a number of residues (e.g. residues 60, 62, 65) the RDC data show markedly lower RDC-order parameters than the REDOR data. One possible explanation could be found in the presence of motion on time scales between the one relevant for solid-state (~10 μs) and solution-state (~10 ms). In fact, there is some experimental evidence for motion in ubiquitin on a time scale of 10 μs (Ban et al. 2011). This microsecond motion was, however, detected there for a small set of residues, and these data do not provide an explanation for all residues for which we observe lower RDC order parameters. Somewhat unexpectedly also, in the RDC data set that matches the REDOR data apparently better (in terms of overall motional amplitude), there are a few residues that have rather large order parameters, i.e. values of S^2^(RDC) exceeding the REDOR order parameters (see Fig. 10a). In these cases the RDC order parameters also exceed the solution-state relaxation-derived value (which is sensitive to sub-nanosecond motion). This is an unphysical situation, as has been noted before (Salmon et al. 2009), and it has been speculated that uncertainties in the relaxation order parameters may be the origin. Although we cannot exclude this possibility, the good match of solution-state relaxation data with REDOR data seems to weaken this argument. An alternative explanation to both the large site-to-site variation of RDC-order parameters, and the unphysically high values might also lie in uncertainties in the determination of the RDC order parameters. Our new dipolar coupling data might be useful as a benchmark for continued development of approaches to analyze RDC data. As has been pointed out previously, the REDOR data might also be compared directly to solution-state order parameters, and differences might be interpreted in terms of ns-μs motion (Chevelkov et al. 2010).

## Conclusions

In this paper, we have provided a detailed analysis of some approaches for determining protein backbone motion on time scales from picoseconds to microseconds, both from the perspective of simulated data, as well as from a rather extensive set of experimental data, including previously unpublished data sets of ^15^N R_1ρ_ data as well as ^1^H–^15^N dipolar couplings.

We have analyzed in detail the protocol to determine one-bond dipolar couplings in deuterated proteins, placing particular emphasis on eliminating possible sources of systematic errors. From our investigations it has become clear that rf mis-settings and inhomogeneities may introduce systematic errors of a few percent, which is a substantial error when seen on the scale of the motional amplitude, 1–S^2^. The REDOR experiment has a significant advantage over other recoupling schemes, namely its reliance on a train of well-separated π pulses. Calibrating such pulses is more straightforward than calibrating rf fields for continuous-irradiation based approaches. Furthermore, phase transients and instability of the rf fields impact these sequences more than the REDOR sequence. The only limitation of the REDOR experiment is its requirement for rather well isolated spin pairs, such as ^1^H–^15^N in deuterated proteins (Schanda et al. 2011b). Our new data, obtained with accurate pulse calibration and consideration of the rf inhomogeneity, also point to systematic offset of a previously published data set, but reveal that the profile of S^2^ values over the sequence is highly reproducible.

We have shown that it is crucial to have such dipole-coupling data for fitting backbone dynamics. Using relaxation data alone generally leads to systematic bias of order parameters, i.e. an overestimation of S^2^. Furthermore, fitting R_1_ and R_1ρ_ or CCR data will essentially always lead to fits that indicate nanosecond motion, even if there is no such motion present. This has been shown by simulations (Fig. 2) and experimental data (Fig. 7). In this sense, the detection of nanosecond motion, that has been claimed in recent studies (Knight et al. 2012; Lewandowski et al. 2010a, b), may be artifactual.

We show that measurements at a single magnetic field strength are sufficient if only the SMF approach is used, and with some reasonable assumptions even an EMF approach may be fitted from single-field measurements.

The findings and protocols that are shown here for ^15^N sites along the backbone likely also apply to other sites, that have so far received less attention, such as methyl side chains (Agarwal et al. 2008; Schanda et al. 2011a), or backbone carbon sites (Asami and Reif 2013). We foresee that for these sites it will be equally important to complement relaxation data with dipolar coupling measurements. Given appropriate labeling, such as recently proposed selective methyl labeling (Agarwal et al. 2008; Schanda et al. 2011a) or random sparse protonation (Asami et al. 2010), the findings reported here for ^15^N amide sites can readily be applied to other backbone and side chain sites. Such studies will allow to provide a comprehensive picture of protein motion, including proteins that are inaccessible to other techniques, such as insoluble or very large protein assemblies.

## Electronic supplementary material

Below is the link to the electronic supplementary material.

Supplementary material 1 (PDF 7122 kb)
