# Supplementary material for: Amplitudes and time scales of picosecond-to-microsecond motion in proteins studied by solid-state NMR: a critical evaluation of experimental approaches and application to crystalline ubiquitin
Source: J Biomol NMR. 2013 Oct 9;57(3):263–80. doi: 10.1007/s10858-013-9787-x (PMC3840295; doi:10.1007/s10858-013-9787-x)
Supplement: Supplementary file 1 — Supplementary material 1 (PDF 7122 kb) [file 10858_2013_9787_MOESM1_ESM.pdf]

Supporting Information

Amplitudes and time scales of picosecond-to-microsecond motion in proteins studied by solid-state NMR: a critical evaluation of erimental approaches and application to crystalline ubiquitin

Jens D. Haller[1,2,3] and Paul Schanda[1,2,3,*]

1 Univ. Grenoble Alpes, Institut de Biologie Structurale (IBS), F-38027 Grenoble, France
2 CEA, DSV, IBS, F-38027 Grenoble, France
3 CNRS, IBS, F-38027 Grenoble, France

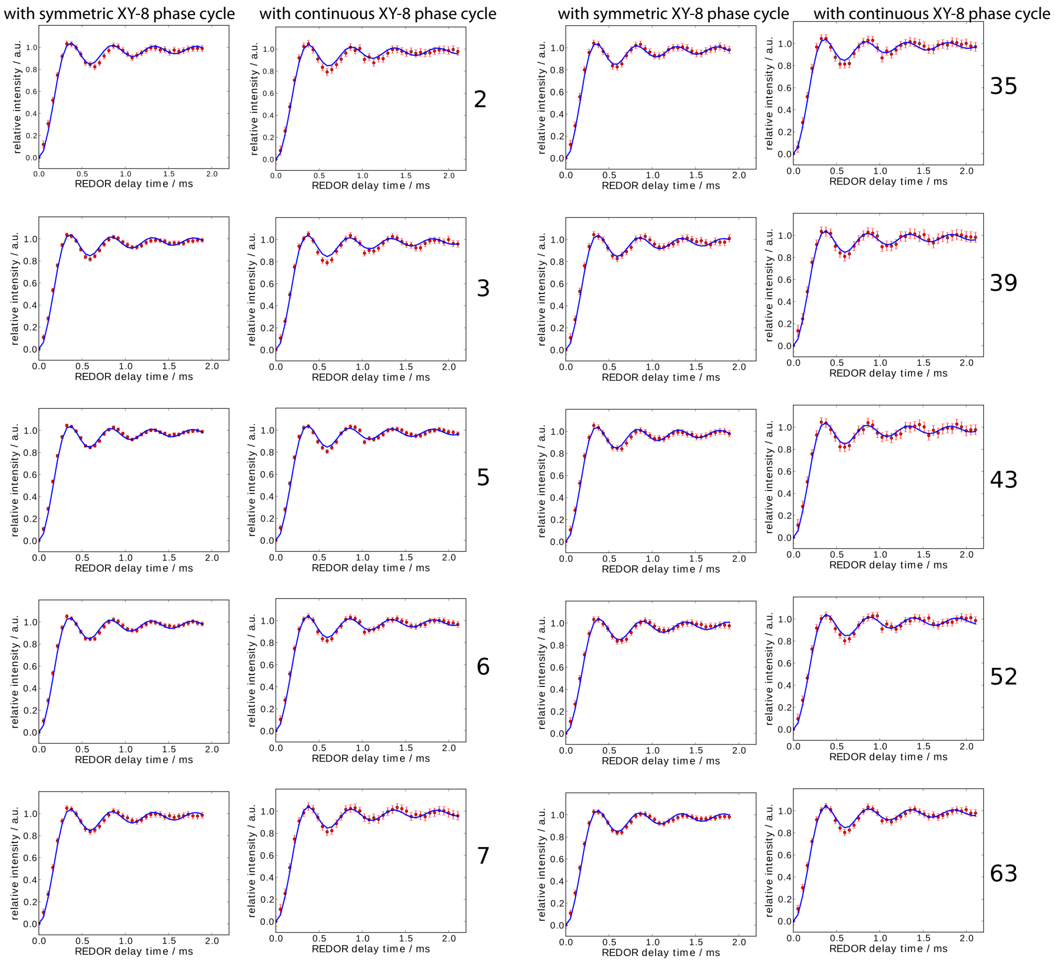

Figure S1.

Representative REDOR curves measured with a pulse sequence that used a mirror-symmetric implementation of the XY-8 phase cycle in the REDOR element (first and third column), i.e. the phases in the second half of the REDOR block are mirrored with respect to the first half, or a continuous phase cycle scheme, where the XY-8 pulse phases are incremented irrespectively of whether the pulse is in the first or second half (second and fourth columns). One can see that the latter implementation results in a somewhat lower first minimum. Curves in the first/second and third/fourth column are for the same residue, and the respective residue numbers are shown on the right of the respective plots.

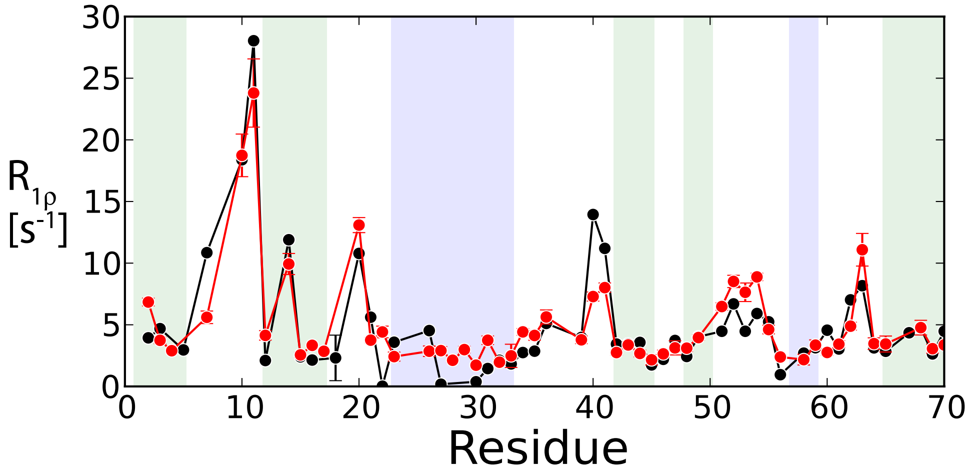

Figure S2:

$R_{1\rho}$ rate constants measured in deuterated, 50% back-exchanged ubiquitin at 15kHz ^{15}N rf field and 39.5kHz MAS (red) are compared to back-calculated values of $R_{1\rho}$. For the latter, all relaxation- and dipolar-coupling data, except for the $R_{1\rho}$ data set, were fit in an EMF model, and $R_{1\rho}$ were calculated for each residue from the four dynamic parameters of the EMF model.

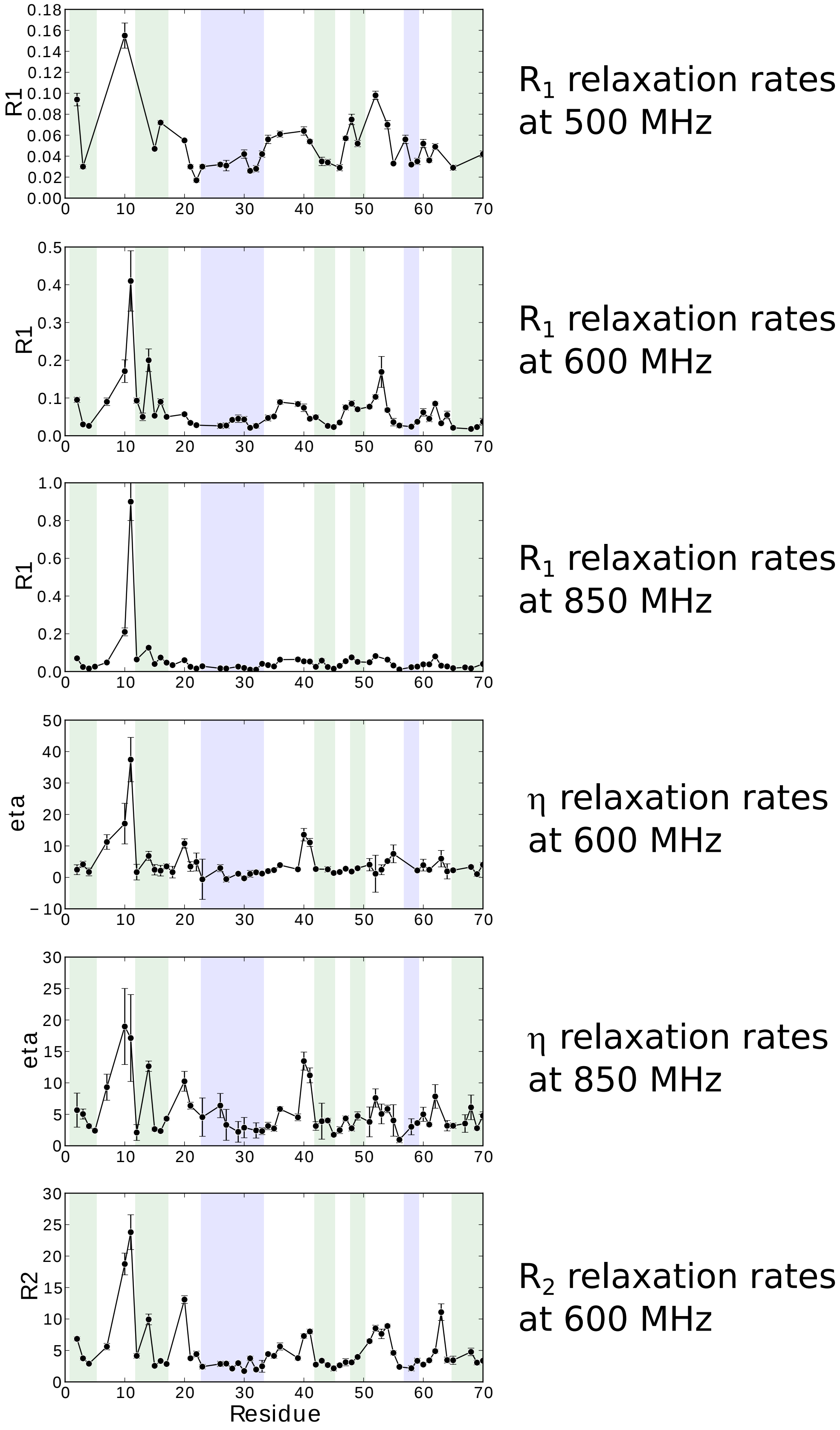

Figure S3.

Experimental relaxation data used in this study for R_1, D/CSA cross-correlated relaxation (η) and R2, derived from $R_{1\rho}$. All relaxation data in [s^{-1}].

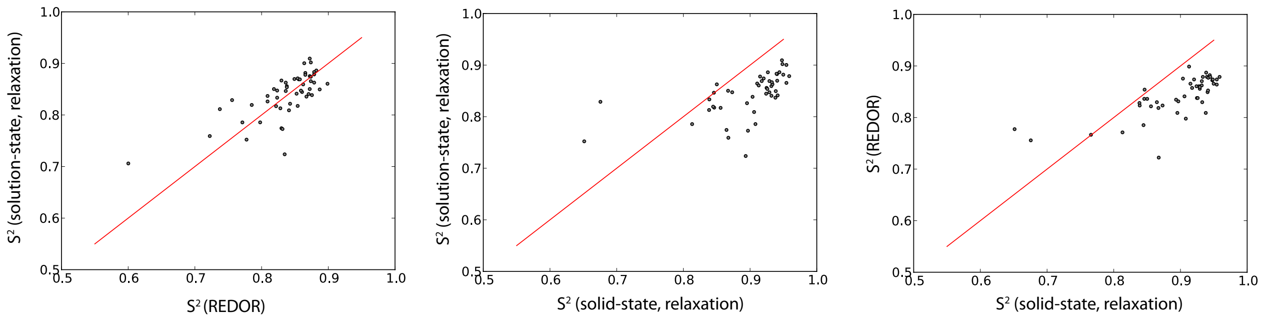

Figure S4:
Correlation plots of S^2 values, derived from REDOR, solid-state relaxation and solution-state relaxation analyses.

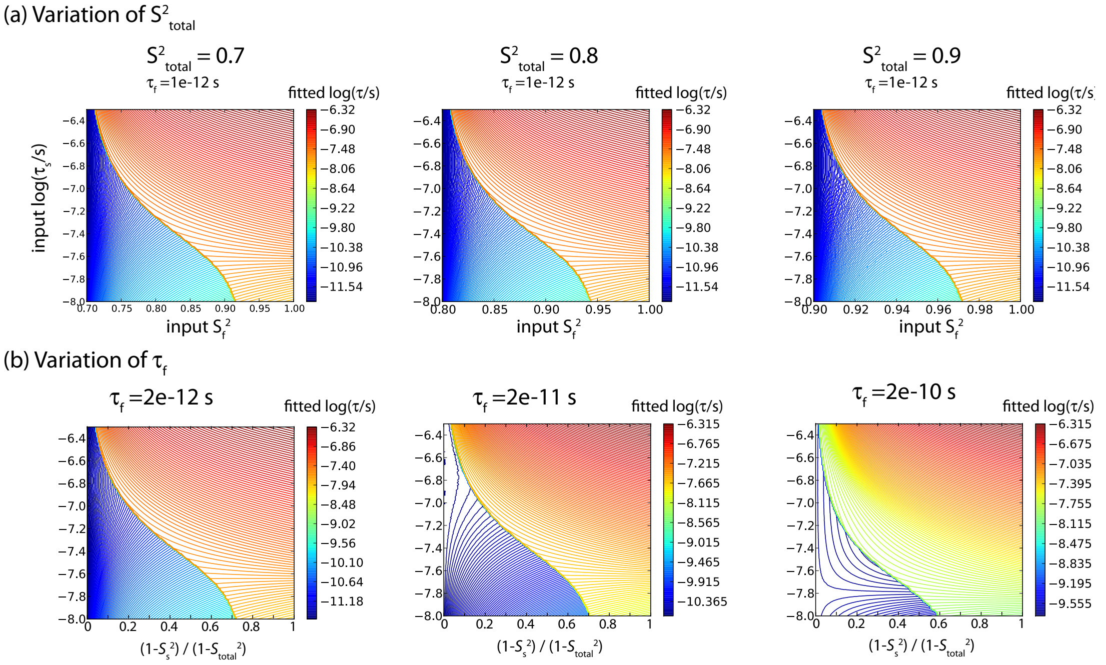

Figure S5:

Fits of EMF-based motional models with the SMF fit, as described in Figure 7. In panel (a), it is shown that the graphs do not depend on the total order parameter of the input EMF model, but only on the relative amount of slow/fast motions. The three graphs, assuming different S^2_{total}, are identical. In (b) we show how the graph changes when the fast-motion correlation time is changed. As expected, if the fast motion gets slower, then even a smaller amplitude of the slow-motion S^2_s will result in the detection of slow motion upon SMF fit.

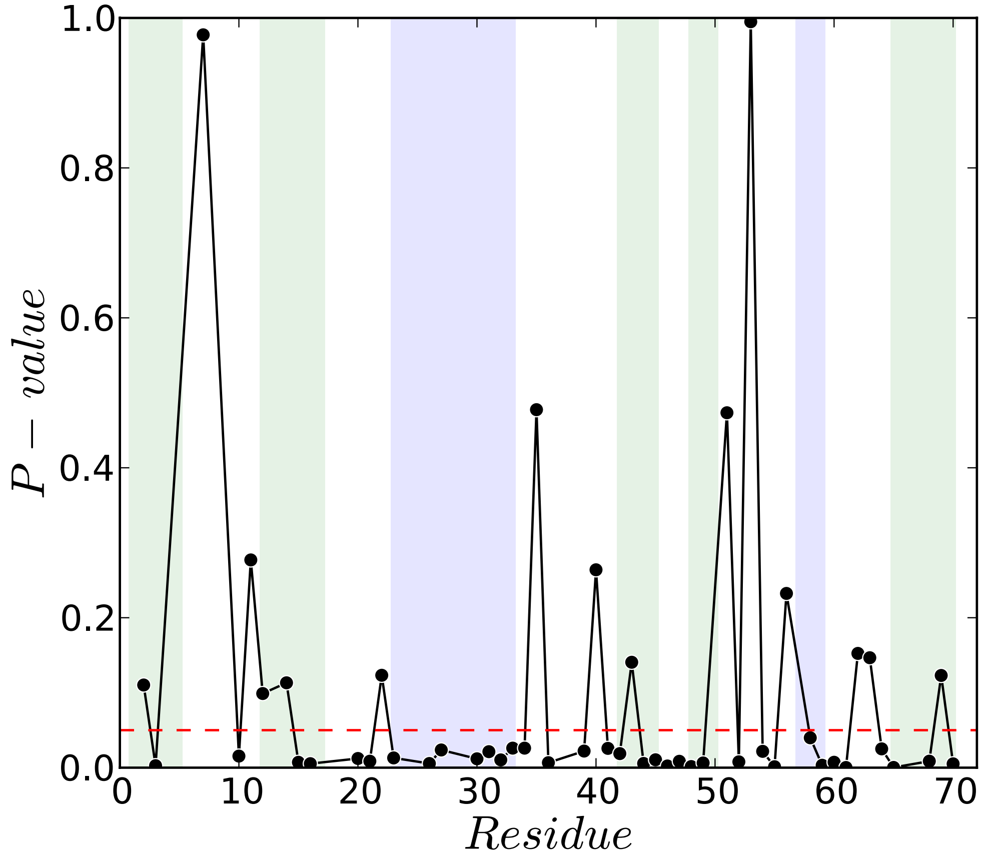

Figure S6.
F-test statistics of EMF vs SMF models. Low P-value indicate acceptance of the more complex EMF model. The threshold is set at 0.05.

Figure S7 (this and the next page):
Investigation of the robustness of SMF and EMF fits when data sets are eliminated.

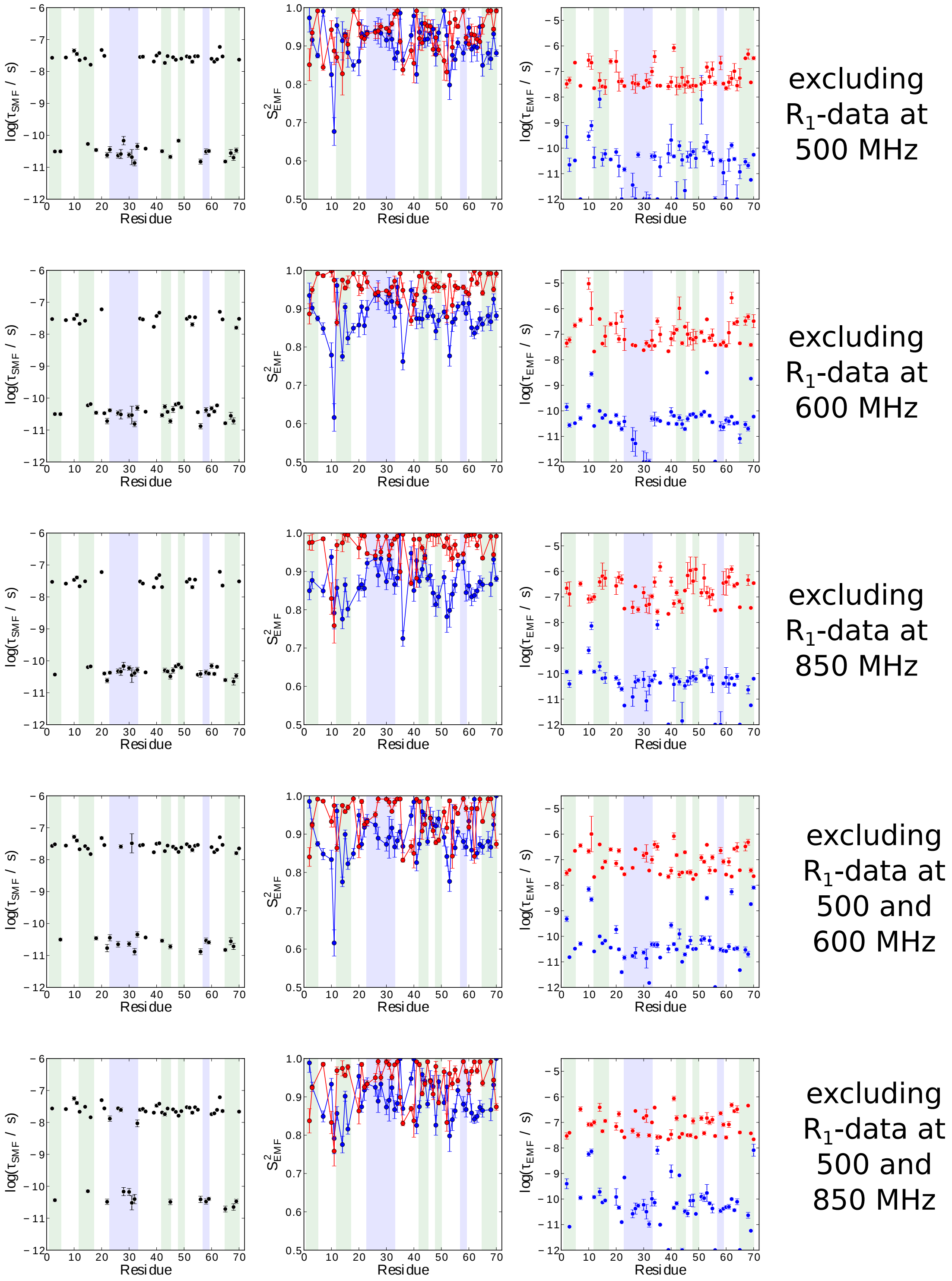

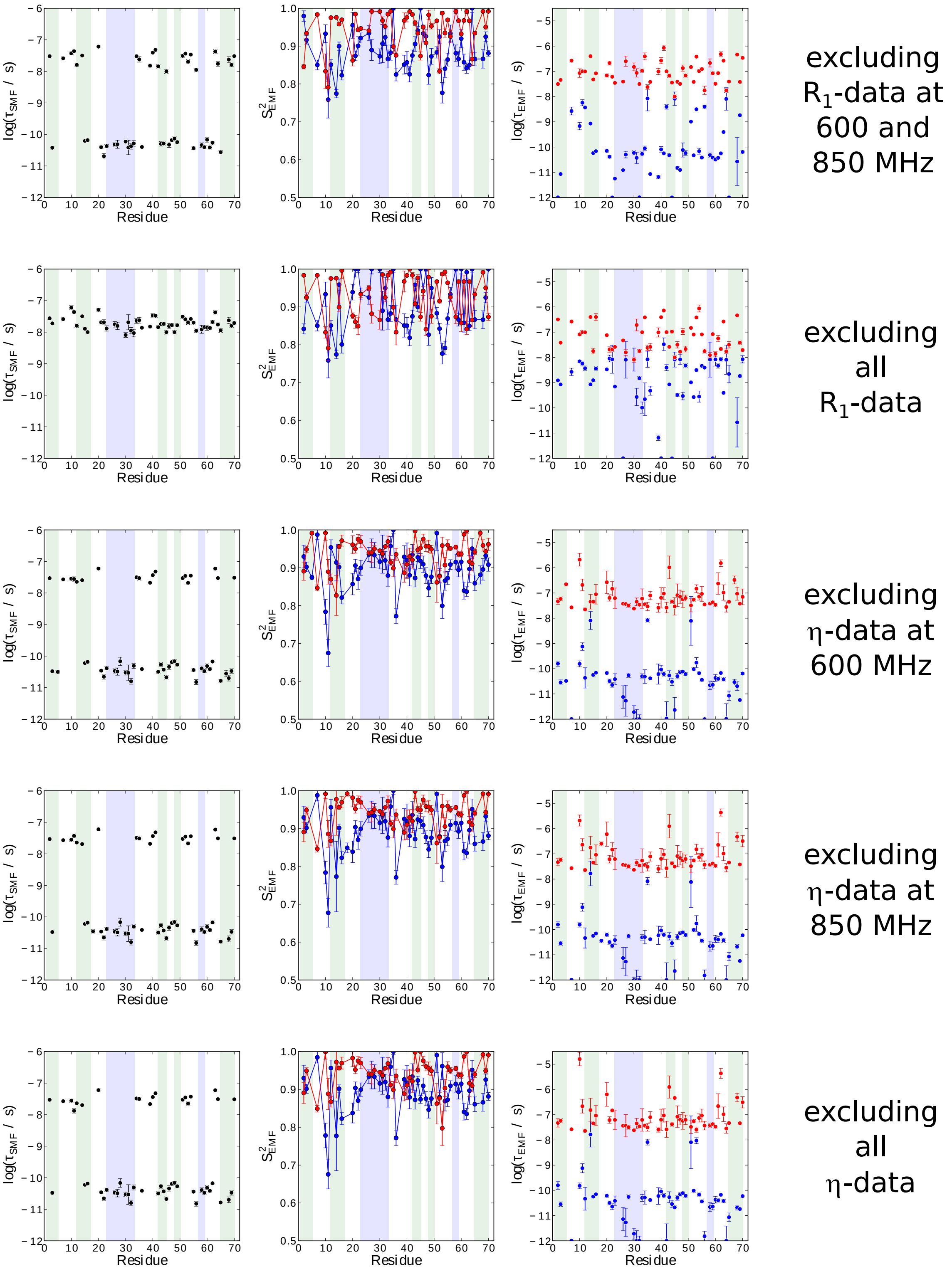

log(τ_SMF / s)
S^2_{EMF}
log(τ_EMF / s)
Residue
excluding R₁-data at 600 and 850 MHz
excluding all R₁-data
excluding η-data at 600 MHz
excluding η-data at 850 MHz
excluding all η-data

Figure S8.
EMF fits with data obtained at a single magnetic field strength, assuming different values of τ_f. See Figure 9 for more description.

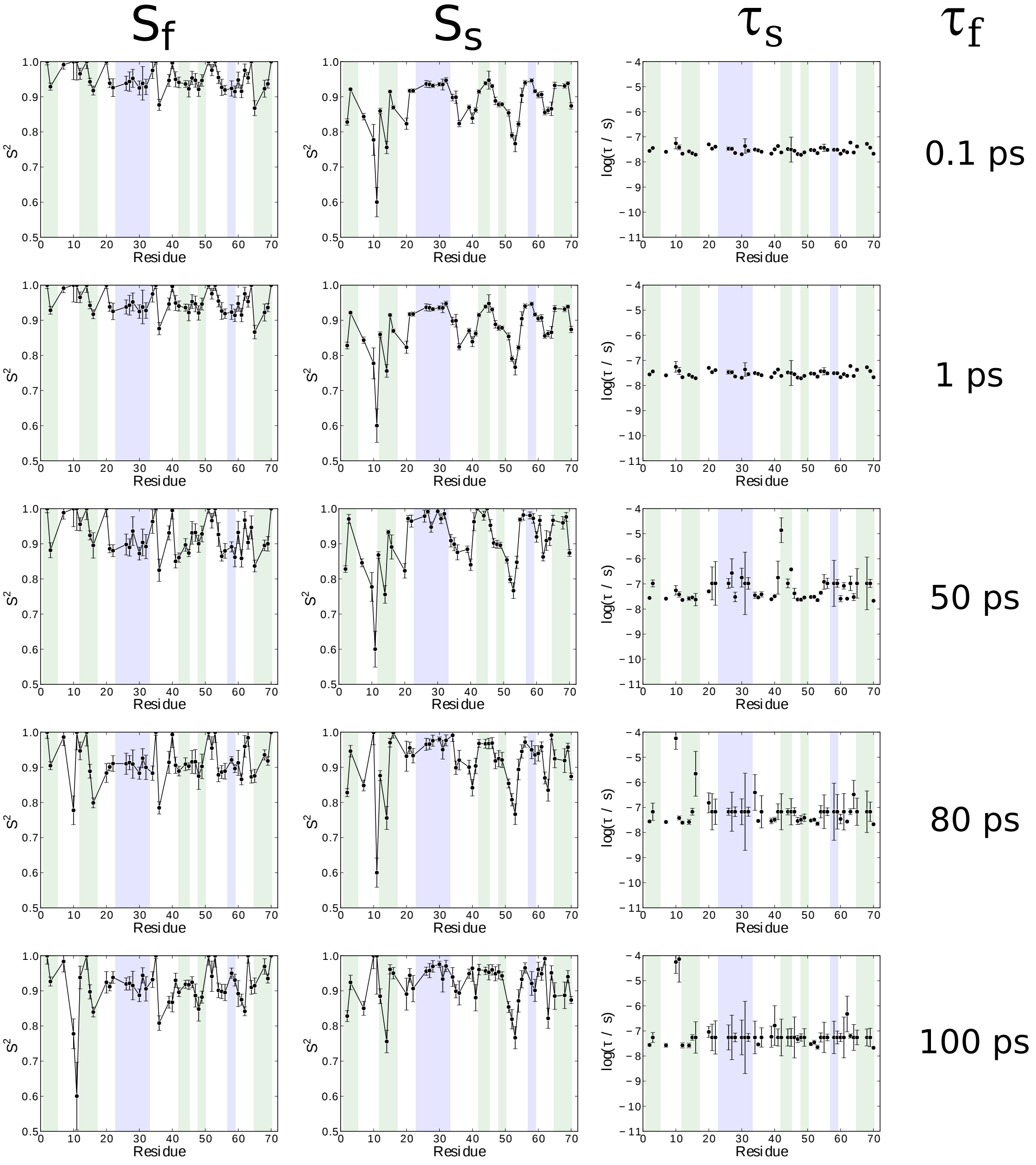

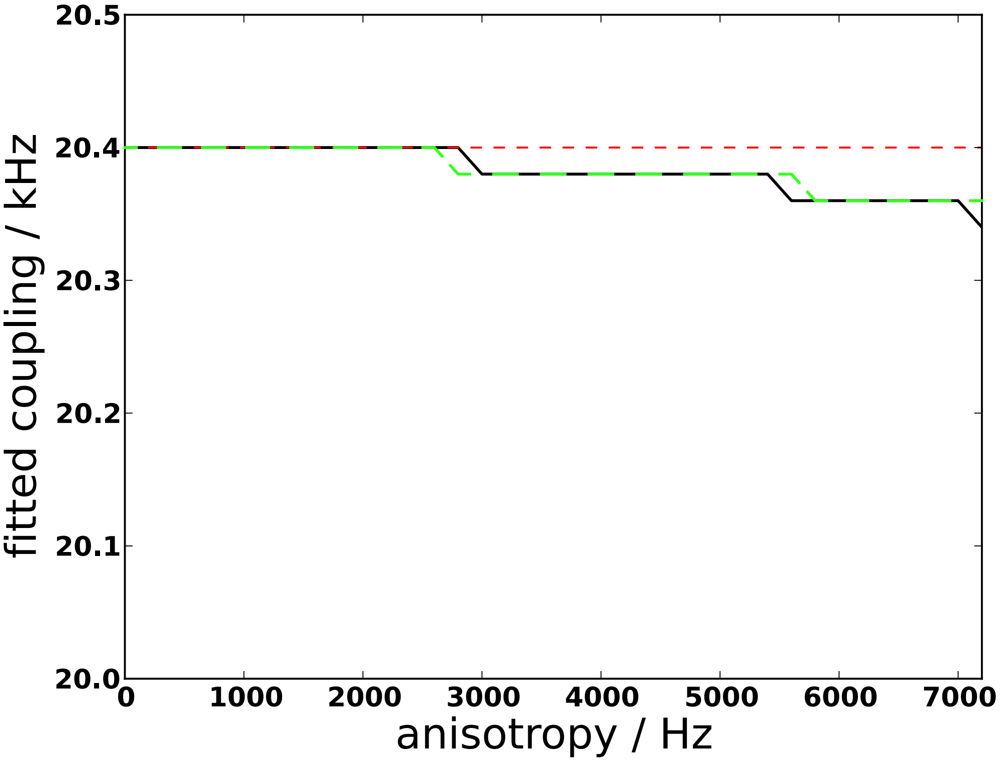

Figure S9. Investigation of the effect of ^{1}H CSA tensors on the fitted NH dipolar coupling in the REDOR experiment, using numerical simulations. The evolution of the ^{15}N coherence undergoing the REDOR recoupling (Figure 3) was fitted using GAMMA. Here, a 2-spin NH spin system was assumed, the NH dipolar coupling was set to 20.4kHz, the ^{15}N CSA tensor was set to σ_z=113ppm ($\Delta\sigma$=170ppm), and the timing was identical to the one used in the main text. The ^{1}H CSA tensor was assumed to have anisotropies from 0 to 7200 Hz, and an asymmetry parameter of either 0.1 (black), or 0.9 (green, dashed). These simulations were then fitted against 2-spin simulations, in an identical manner as the experimental data were fitted, using a grid search procedure (hence then discontinuous behavior). These data reveal that the ^{1}H CSA tensor has essentially no effect on the fitted dipolar couplings in the REDOR experiment.

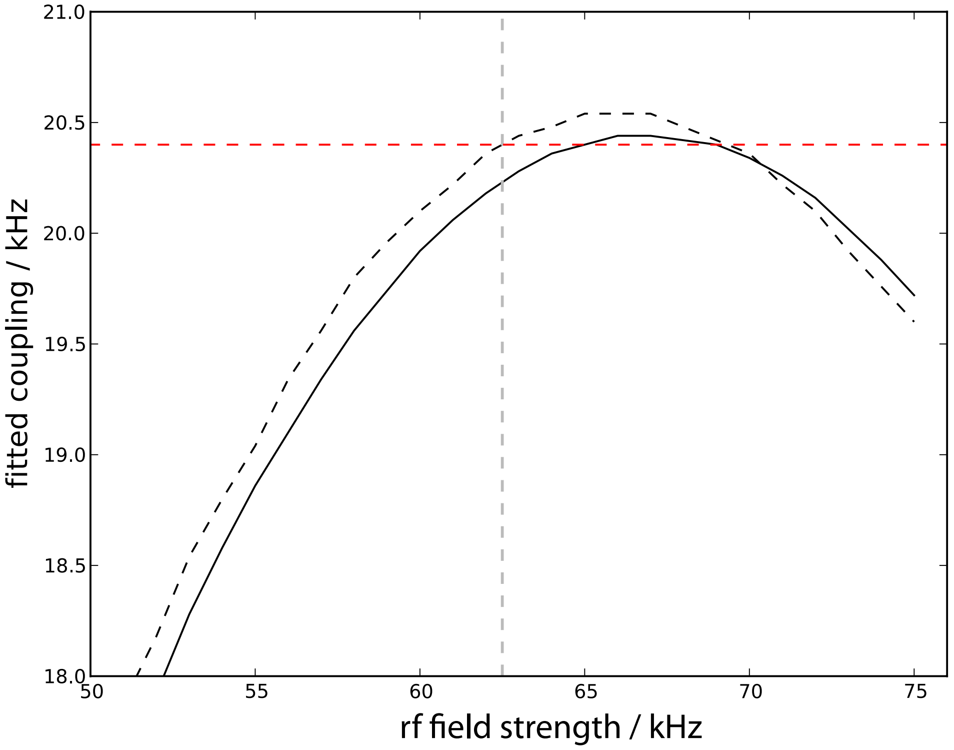

Figure S10. Investigation of the effect of rf mis-setting and rf inhomogeneity on dipolar couplings extracted from the REDOR experiment. This Figure is analogous to Figure 3e in the main text. The only difference is that the pulse duration was set to 8μs, thus corresponding to a nominal rf field of 62.5kHz. The delay τ in Figure 3a was set to 4.5μs, i.e. the minimum delay between subsequent pulses in the REDOR train was 0.5μs, as in the other experiments and simulations. The dashed line shows the situation where the rf field was homogeneous, i.e. applied at one single value. The solid line assumes rf inhomogeneity. Here, we assumed that the rf inhomogeneity is proportional to the applied field, i.e. the rf field distribution that has been obtained experimentally at approximately 100kHz rf field (Figure 3f) was scaled by 0.625 (=62.5/100), and this rf field distribution was then used to explicitly sum REDOR curves obtained with different rf fields.

These data show that the effect of mis-setting and rf inhomogeneity is very similar in the case that 5μs pulses or 8μs pulse are applied.

Table S1:

Experimental dipolar-coupling derived squared order parameters. These data, shown in Figure 5 of the main text, were obtained from the REDOR experiment. The REDOR curves were fitted against 2-spin simulations in GAMMA, that take into account the finite pulse length and phase cycle, assuming 180° pulses. The rf inhomogeneity was taken into account by scaling the fitted D couplings by 1.015. Values reported in this table are squared order parameters derived as $S^2=(\delta_D/\delta_{D,rigid})^2$. , where δ_D is the experimental dipole coupling obtained as above described, and $\delta_{D,rigid}$ =22954Hz, i.e. a bond length of 1.02Å. Error bars were derived from Monte Carlo simulations, where synthetic data sets, obtained from adding random error, according to two times the spectral noise, to the fitted curve. 500 such data sets were generated and the standard deviation over the fitted values is reported.

Residue number	S^2	error bar
2	0.828	0.013
3	0.856	0.010
5	0.867	0.008
6	0.872	0.011
7	0.836	0.013
10	0.777	0.026
11	0.600	0.025
12	0.830	0.013
14	0.756	0.011
15	0.862	0.009
16	0.798	0.011
18	0.843	0.013
20	0.823	0.019
21	0.861	0.011
22	0.849	0.024
23	0.872	0.012
26	0.879	0.017
27	0.882	0.026
28	0.887	0.026
30	0.866	0.019
31	0.877	0.051
32	0.879	0.022
33	0.852	0.023
34	0.875	0.021
35	0.899	0.019
36	0.722	0.015
39	0.823	0.015
40	0.836	0.023
41	0.819	0.020
42	0.861	0.014
43	0.871	0.015
44	0.879	0.009
45	0.874	0.011
46	0.887	0.019
47	0.841	0.017
48	0.809	0.018
49	0.831	0.016

50	0.872	0.015
51	0.854	0.015
52	0.771	0.014
53	0.767	0.025
54	0.785	0.016
55	0.838	0.014
56	0.864	0.011
57	0.854	0.028
58	0.874	0.020
59	0.838	0.016
60	0.857	0.020
61	0.830	0.017
62	0.835	0.013
63	0.822	0.012
64	0.866	0.017
65	0.809	0.018
67	0.874	0.023
68	0.859	0.025
69	0.879	0.011
70	0.874	0.009
71	0.737	0.014

Table S2:

Experimental data of R_2, as derived from $R_{1\rho}$ measurements, as described in the Materials and Methods section.

Residue number	R_2 / s^{-1}	error bar / s-1
2	6.84	0.31
3	3.74	0.27
4	2.90	0.13
7	5.61	0.51
10	18.74	1.73
11	23.80	2.77
12	4.14	0.38
14	9.93	0.85
15	2.56	0.22
16	3.33	0.15
17	2.85	0.13
20	13.09	0.61
21	3.76	0.21
22	4.42	0.47
23	2.42	0.37
26	2.85	0.42
27	2.90	0.33
28	2.12	0.20
29	2.99	0.27
30	1.72	0.13
31	3.75	0.35
32	1.97	0.23
33	2.48	0.94
34	4.42	0.27
35	4.13	0.39
36	5.63	0.57
39	3.78	0.31
40	7.29	0.37
41	8.01	0.38
42	2.75	0.21
43	3.36	0.26
44	2.68	0.27
45	2.16	0.42
46	2.64	0.30
47	3.13	0.57
48	3.10	0.27
49	3.96	0.23
51	6.48	0.30
52	8.52	0.50
53	7.64	0.75
54	8.88	0.30
55	4.61	0.35
56	2.39	0.17
58	2.17	0.43
59	3.35	0.42
60	2.75	0.23
61	3.43	0.28
62	4.89	0.32

63	11.09	1.32
64	3.47	0.47
65	3.44	0.65
68	4.77	0.59
69	3.06	0.36
70	3.37	0.19

Table S3.

Fitted parameters in the model-free fits, using all available data sets. The number of data points used is indicated for each residue.

Residue	chi2(SMF)	chi2(EMF)	# available data	S2(SMF)	S2f(EMF)	S2s(EMF)	tau(SMF)	tauf(EMF)	taus(EMF)
2	2.11E+01	4.93E+00	7	0.83	0.93	0.89	8.28E-01	1.57E-10	4.67E-08
3	2.59E+02	5.46E+00	7	0.86	0.90	0.95	8.56E-01	2.87E-11	5.88E-08
5	1.00E+02	1.50E-05	3	0.87	0.87	0.99	8.67E-01	3.29E-11	2.22E-07
7	1.37E+01	1.26E+01	6	0.84	0.99	0.84	8.36E-01	1.00E-12	2.75E-08
10	8.31E+01	5.57E+00	7	0.78	0.78	0.99	7.77E-01	1.56E-10	2.08E-06
11	8.49E+01	2.21E+01	6	0.60	0.68	0.89	6.00E-01	7.61E-10	2.21E-07
12	2.92E+01	2.82E+00	6	0.83	0.95	0.87	8.30E-01	4.31E-11	2.22E-08
14	4.53E+01	4.99E+00	6	0.76	0.91	0.83	7.56E-01	8.21E-09	4.46E-08
15	2.47E+02	9.99E+00	7	0.86	0.90	0.96	8.62E-01	5.65E-11	4.53E-08
16	5.88E+02	1.97E+01	7	0.80	0.82	0.97	7.98E-01	6.92E-11	8.90E-08
18	8.03E-01	1.31E-05	3	0.84	0.85	0.99	8.43E-01	3.60E-11	2.51E-07
20	8.12E+01	4.67E+00	7	0.82	0.84	0.98	8.24E-01	6.20E-11	5.56E-07
21	4.53E+02	2.07E+01	7	0.86	0.91	0.95	8.61E-01	3.24E-11	6.38E-08
22	1.02E+02	1.22E+01	6	0.85	0.87	0.98	8.49E-01	2.27E-11	1.47E-07
23	4.58E+01	5.94E-01	6	0.87	0.90	0.97	8.72E-01	3.84E-11	6.22E-08
26	1.02E+02	3.49E+00	7	0.88	0.94	0.94	8.79E-01	7.54E-12	3.80E-08
27	8.82E+01	7.79E+00	7	0.88	0.94	0.94	8.82E-01	4.34E-12	3.49E-08
28	1.12E+02	5.69E-06	3	0.89	0.93	0.95	8.87E-01	5.51E-11	3.19E-08
30	2.24E+02	1.26E+01	7	0.87	0.92	0.95	8.66E-01	1.00E-12	2.34E-08
31	1.43E+02	3.04E+00	6	0.88	0.93	0.94	8.77E-01	1.00E-12	4.43E-08
32	1.25E+02	6.48E+00	7	0.88	0.92	0.96	8.79E-01	1.00E-12	3.46E-08
33	3.42E+01	8.85E-01	6	0.85	0.88	0.97	8.53E-01	4.95E-11	5.79E-08
34	7.48E+01	7.03E+00	7	0.88	0.96	0.92	8.77E-01	4.76E-11	3.53E-08
35	2.04E+01	8.84E+00	6	0.90	0.99	0.91	8.99E-01	1.00E-12	2.79E-08
36	5.43E+02	2.10E+01	7	0.72	0.77	0.94	7.22E-01	4.14E-11	7.90E-08
39	7.09E+01	1.56E+00	6	0.82	0.93	0.89	8.24E-01	6.09E-11	2.58E-08
40	8.68E+01	3.44E+01	7	0.84	0.92	0.91	8.37E-01	9.44E-11	6.54E-08
41	2.52E+02	2.35E+01	7	0.82	0.88	0.93	8.20E-01	6.21E-11	9.60E-08
42	2.65E+02	4.96E+00	6	0.86	0.94	0.92	8.61E-01	1.00E-12	2.70E-08
43	1.73E+02	3.15E+00	5	0.87	0.87	1.00	8.71E-01	5.39E-11	1.04E-06
44	2.42E+02	8.36E+00	7	0.88	0.93	0.95	8.79E-01	3.04E-11	4.55E-08
45	7.82E+01	8.22E-01	6	0.87	0.92	0.95	8.74E-01	2.18E-12	2.95E-08
46	1.19E+02	2.25E+00	7	0.89	0.91	0.98	8.87E-01	5.04E-11	8.10E-08
47	2.26E+02	1.03E+01	7	0.84	0.88	0.96	8.41E-01	7.20E-11	7.09E-08
48	2.40E+02	3.46E+00	7	0.81	0.84	0.96	8.09E-01	7.64E-11	5.44E-08
49	3.84E+02	1.43E+01	7	0.83	0.88	0.95	8.31E-01	6.11E-11	6.30E-08
51	3.60E+00	1.55E+00	6	0.85	0.99	0.86	8.54E-01	7.80E-09	3.23E-08
52	1.26E+02	5.37E+00	7	0.77	0.88	0.88	7.72E-01	9.61E-11	5.45E-08
53	5.29E+00	5.11E+00	5	0.77	0.80	0.96	7.67E-01	1.73E-10	1.48E-07
54	3.13E+02	2.62E+01	7	0.79	0.86	0.91	7.86E-01	6.58E-11	7.08E-08
55	1.85E+02	2.37E+00	7	0.84	0.87	0.96	8.38E-01	3.63E-11	9.25E-08
56	2.13E+02	1.06E+01	5	0.86	0.91	0.95	8.64E-01	1.00E-12	3.51E-08
58	6.88E+01	2.71E+00	6	0.87	0.92	0.95	8.74E-01	2.20E-11	3.82E-08
59	2.75E+02	6.55E+00	7	0.84	0.89	0.94	8.38E-01	2.27E-11	4.23E-08
60	1.80E+02	7.33E+00	7	0.86	0.92	0.94	8.57E-01	4.31E-11	3.42E-08
61	2.97E+02	1.19E+00	7	0.83	0.84	0.99	8.30E-01	3.99E-11	2.43E-07
62	2.89E+02	4.25E+01	6	0.83	0.84	1.00	8.35E-01	6.76E-11	1.53E-06

63	2.92E+01	5.79E-01	5	0.82	0.90	0.92	8.22E-01	3.75E-11	1.02E-07
64	5.90E+01	1.47E+00	6	0.87	0.95	0.91	8.66E-01	1.00E-12	2.79E-08
65	1.20E+02	6.06E-01	7	0.81	0.86	0.94	8.09E-01	8.47E-12	4.48E-08
67	6.35E+00	1.60E-05	3	0.87	0.88	0.99	8.74E-01	2.93E-11	3.29E-07
68	1.07E+02	9.19E-01	6	0.86	0.87	0.99	8.59E-01	2.08E-11	4.80E-07
69	1.68E+02	2.35E+00	5	0.88	0.93	0.94	8.79E-01	5.73E-12	3.74E-08
70	2.98E+02	9.66E+00	7	0.87	0.88	0.99	8.74E-01	5.92E-11	3.25E-07
